# Subunit nanovaccine elicited T cell functional activation controls *Trypanosoma cruzi* mediated maternal and placental tissue damage and improves pregnancy outcomes in mice

**DOI:** 10.1038/s41541-023-00782-z

**Published:** 2023-12-16

**Authors:** Lizette Elaine Rios, Nandadeva Lokugamage, Subhadip Choudhuri, Imran Hussain Chowdhury, Nisha Jain Garg

**Affiliations:** 1 Department of Microbiology and Immunology, University of Texas Medical Branch (UTMB), Galveston, TX USA; 2 Department of Biochemistry and Molecular Biology, UTMB, Galveston, TX USA; 3 Institute for Human Infections and Immunity (IHII), UTMB, Galveston, TX USA; 4 Sealy Institute for Vaccine Sciences (SIVS), UTMB, Galveston, TX USA

**Keywords:** Infectious diseases, Vaccines, Infection

## Abstract

This study investigated a candidate vaccine effect against maternal *Trypanosoma cruzi (Tc)* infection and improved pregnancy outcomes. For this, *TcG2* and *TcG*4 were cloned in a nanoplasmid optimized for delivery, antigen expression, and regulatory compliance (nano2/4 vaccine). Female C57BL/6 mice were immunized with nano2/4, infected (*Tc* SylvioX10), and mated 7-days post-infection to enable fetal development during the maternal acute parasitemia phase. Females were euthanized at E12–E17 (gestation) days. Splenic and placental T-cell responses were monitored by flow cytometry. Maternal and placental/fetal tissues were examined for parasites by qPCR and inflammatory infiltrate by histology. Controls included age/immunization-matched non-pregnant females. Nano2/4 exhibited no toxicity and elicited protective IgG2a/IgG1 response in mice. Nano2/4 signaled a splenic expansion of functionally active CD4+ effector/effector memory (Tem) and central memory (Tcm) cells in pregnant mice. Upon challenge infection, nano2/4 increased the splenic CD4+ and CD8+T cells in all mice and increased the proliferation of CD4+Tem, CD4+Tcm, and CD8+Tcm subsets producing IFNγ and cytolytic molecules (PRF1, GZB) in pregnant mice. A balanced serum cytokines/chemokines response and placental immune characteristics indicated that pregnancy prevented the overwhelming damaging immune response in mice. Importantly, pregnancy itself resulted in a significant reduction of parasites in maternal and fetal tissues. Nano2/4 was effective in arresting the *Tc*-induced tissue inflammatory infiltrate, necrosis, and fibrosis in maternal and placental tissues and improving maternal fertility, placental efficiency, and fetal survival. In conclusion, we show that maternal nano2/4 vaccination is beneficial in controlling the adverse effects of *Tc* infection on maternal health, fetal survival, and pregnancy outcomes.

## Introduction

*Trypanosoma cruzi (Tc*) is the etiological agent for Chagas disease (CD). The prolonged burden of CD primarily occurs in Latin America, Mexico, and USA^1^. The CD is recognized as a major health issue in other countries including Japan, Canada, Australia, and Europe due to the movement of infected individuals from endemic to non-endemic countries^2^. Further, congenital CD has surfaced as a major health issue in both endemic and non-endemic nations. The World Health Organization estimates that ~2-million women of childbearing age are infected^3^, and give birth to >18,000 infected infants every year^4^. Maternal *Tc* infection increases the risk of pre-term birth, low birth weight, and stillbirth of neonates^5^. Neonates infected via vertical transmission of *Tc* are projected to be at risk of developing hepatosplenomegaly, myocarditis, or meningoencephalitis during the first few years of life, and continue to be at risk of developing cardiomyopathy, and neurological and digestive disorders as they get older^6^. A recent study in Bolivia found that 29% of the neonates born with *Tc* infection developed clinical manifestations of acute cardiac disease^7^. It was proposed that congenital *Tc* infection may be a factor for the early on-set of symptomatic heart disease that is observed in people infected by other routes. Others have documented that >30% of young children exposed to *Tc* may develop neurological complications accompanied by behavioral and cognitive impairments as young adults^8^. Some studies indicate that fetuses exposed to maternal infection during gestation are born with low birth weight and altered immune capacity^9^; whether these offspring are at risk of CD-associated morbidities as young adults are not studied.

The currently accessible medicines for treating *Tc* infection are nifurtimox and benznidazole. These drugs are contraindicated for pregnant women because of their potential teratogenic effects^10^. Vaccines for preventing *Tc* infection in humans are not currently available. Yet, the subunit vaccines encoding GP90, TC52, CRP, ASP2, or other *Tc* antigens that exhibit low homology to host proteins have been shown to generate a degree of protection from CD in experimental studies (reviewed in refs. ^4,11^). During the last decade, our team has identified >11 potential vaccine candidates by an unbiased bioinformatics approach along with biological tests^12,13^. Of these, two candidates exhibited repeated efficacy in multiple animal models. These antigens, TcG2 and TcG4, are highly conserved (92–99%) in TcI-TcVI lineages, expressed in infective/intracellular stages of *Tc*, and consist of epitopes presented by MHC alleles of mice, dogs, and humans. After testing a variety of vaccine formulations, delivery vehicles, and adjuvants, we found that TcG2 and TcG4-encoding DNA vaccine is simplest in design and cost to obtain >88% control of tissue parasites in mice and dogs^14–18^.

Next-generation vectors, known as nano eukaryotic expression plasmids, have been developed to enable efficient, secure, and cost-effective gene delivery. Nanoplasmids commonly use synthetic eukaryotic mRNA leaders and terminators to minimize the similarity between the vector DNA sequence and the human genome and decrease the possibility of integration into the host genome by homologous recombination. In order to eliminate the need for antibiotics in the selection and amplification of recombinant DNA, certain nanoplasmids incorporate RNA-OUT as an alternative method^19^. Additionally, they employed an R6K-derived mini-origin (300 bp) and a well-designed SV40-CMV-HTLV-1 R chimeric promoter-intron to achieve sustained and elevated expression of the target genes within mammalian host cells^19^. Thus, nanoplasmids provide a refined delivery framework that enhances the expression of cloned antigens at an affordable cost, and their design adheres to the established regulatory compliance guidelines. We have cloned TcG2 and TcG4 genes into an NTC9385R-MCS nanoplasmid to generate the nano2/4 vaccine^20^.

In this study, we aimed to determine how anti-*Tc* immunity is altered in pregnancy and evaluate the protective efficacy of the nano2/4 vaccine against maternal *Tc* infection and its adverse effects on developing fetuses. For this, C57BL/6 mice were immunized with two doses of nano2/4 (or empty vector) before challenge infection with *Tc* and mated in the early stages of acute infection. Non-pregnant mice (infected or vaccinated/infected) were included as controls so that we can study the effects of pregnancy on anti-*Tc* immunity and immunogenic potential of the candidate vaccine. We examined if nano2/4 differently modulated the systemic and placental CD4^+^ and CD8^+^ T-cell immunity against *Tc* in pregnant (vs. virgin) mice, and whether the nano2/4-induced immunity was effective in arresting the *Tc*-induced pathogenesis in maternal and fetal tissues.

## Results

### Chagas vaccine, challenge, and pregnancy model

**Fig. 1:**
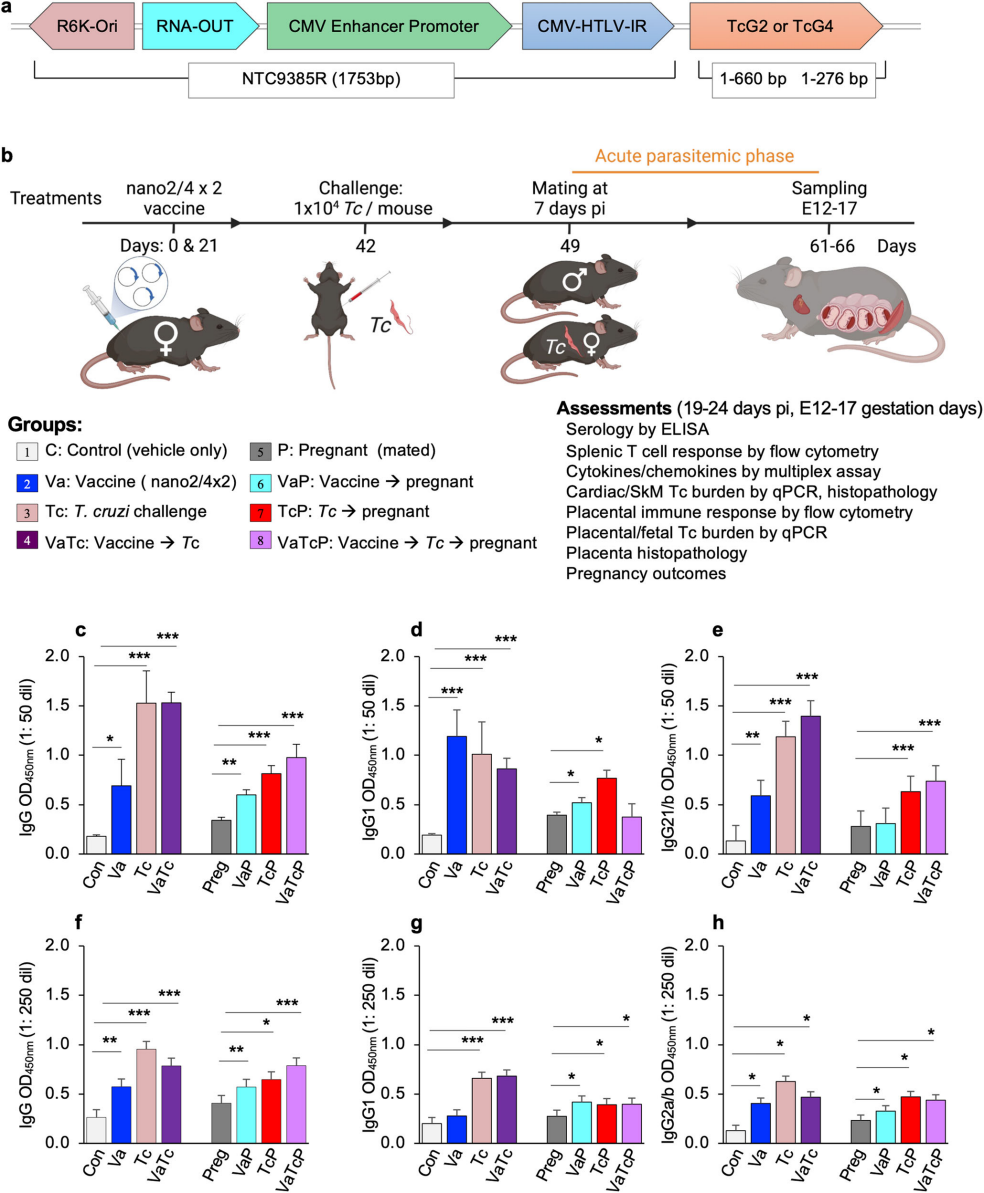
Vaccine-induced antibody response in pregnant mice. **a** Nano vaccine constructs. The cDNAs for TcG2 (1–660 bp) and TcG4 (1–276 bp) were cloned in NTC9385R-MCS plasmid at the SalI/BglII restriction sites. The minimal-size bacterial backbone of the plasmid consists of R6K origin of replication, RNA-OUT selection marker for antibiotic-free amplification, and a chimeric promoter intron (CMV-HTLV-IR) downstream of CMV enhancer-promoter to allow for high levels of target gene expression. **b** Immunization, challenge, and pregnancy model (created using biorender.com). C57BL/6, female mice (6–8 weeks of age) were intramuscularly immunized with two doses of TcG2/TcG4-encoding nanovaccine (referred to as nano2/4) at day 0 and day 21. Challenge infection with *Tc* (SylvioX10) was carried out by intraperitoneal injection of 10,000 parasites per mouse on day 42. When mated, females were housed with males on day 49 (equivalent to 7 days post-infection). Groups: (1) vehicle-only controls (C), (2) nano2/4 vaccine (Va), (3) *Tc* only (Tc), (4) nano2/4 followed by *Tc* (VaTc), (5) pregnant (P), (6) nano2/4 vaccination before pregnancy (VaP), (7) *Tc* before pregnancy (TcP), (8) nano2/4 followed by *Tc* and pregnancy (VaTcP). Mice were euthanized at 61–66 days corresponding to 19–24 days post-infection and E12–17 gestation days for various assessments. **c**–**h** Antibody response. An ELISA was performed to examine the parasite-specific IgG (**c**, **f**), IgG1 (**d**, **g**), and IgG2a/b (**e**, **h**) antibody response in all groups. Sera samples were used at 1: 50 (**c**–**e**) and 1: 250 (**f**–**h**) dilutions. Data are presented as mean values ± SEM derived from duplicate observations per sample (*n* = 6–10 mice per group). Significance was calculated by Students’ unpaired *t*-test with or without Welch’s correction or Mann–Whitney *U*-test and *p*-values of *p* < 0.05, *p* < 0.01, and *p* < 0.001 are annotated with one, two, and three symbols.

Previously, we have shown that mice infected with *Tc* trypomastigotes (SylvioX10 isolate) exhibit acute parasitemia for 7–40 days post-infection (pi). Pathologic inflammatory infiltrate, fibrosis, and oxidative stress can be seen during acute infection, and left ventricular dysfunction is noted at approximately 6 months post-infection^21^. In a recent study, we showed that pregnant Balb/c female mice with acute or chronic *Tc* infection had delayed and lower fertility rates. Offspring born to *Tc*-infected dams showed signs of vertical transmission and had a reduced survival rate, lower birth weight, as well as increased inflammation, necrosis, and fibrosis in their cardiac and brain tissues^22^. Building on these established models, we have examined the efficacy of a two-component DNA vaccine (referred to as nano2/4) against acute *Tc* infection during pregnancy. Our intent was to determine if the nano2/4 vaccine was immunogenic in pregnancy and if it protected pregnant dams from *Tc*-induced pathogenesis. Age and vaccination-matched non-pregnant groups were included in the study to capture how pregnancy changes the host response to *Tc* infection and vaccination. Schematics of the nanovaccine design and the experimental plan are presented in Fig. 1a, b. Briefly, female mice were immunized with two doses of nano2/4 vaccine at day 0 and day 21 and challenge infection with *Tc* was performed on day 42. When mated, females were housed with males on day 7 post-infection. Mice were euthanized at 19–24 days post-infection ( = E12–17 gestation days) for various assessments. No changes in the physical and clinical well-being were observed after vaccination, thus suggesting that the vaccine is safe to deliver in both pregnant and non-pregnant mice.

### Antibody response in nano2/4 immunized pregnant mice (±*Tc*)

We monitored if nano2/4 elicits anti-*Tc* antibody response and whether it is enhanced by challenge infection in pregnant mice. For this, anti-parasite IgG, IgG1, and IgG2a levels were first analyzed using sera samples at 1: 50 dilutions. These data showed that anti-*Tc* IgG, IgG1, and IgG2a levels were increased by 2.9–7.5-fold, 3.5–5.2-fold, and 3.5–9.5-fold, respectively, in non-pregnant groups given vaccine and/or *Tc* infection; and by 34–185%, 0–95%, and 10.5–164%, respectively, in pregnant groups given vaccine and/or *Tc* infection (Fig. 1c–e, *p* < 0.05–0.001). Pregnant mice responded to vaccine and/or infection with lower levels of IgG, IgG1, and IgG2a than was observed in matched non-pregnant groups. Yet, both pregnant and non-pregnant vaccinated/infected mice elicited a higher IgG2a/IgG1 ratio than the matched vaccinated-only or infected-only groups. A similar pattern of IgG, IgG1, and IgG2a response was observed when sera samples were analyzed at 1: 250 dilutions (Fig. 1f–h, *p* < 0.05–0.001). Considering that IFN-γ (Th1 cytokine) and IL-4 (Th2 cytokine) are known to induce isotype switching to IgG2a and IgG1, respectively^23,24^, and IgG2a antibodies are associated with protection from parasitic infections^25,26^, these data suggest that nano2/4 enhances the antibody switch to protective isotype against *Tc* infection in both pregnant and non-pregnant mice.

### Splenic T-cell response to nano2/4 immunization in pregnant mice

**Fig. 2:**
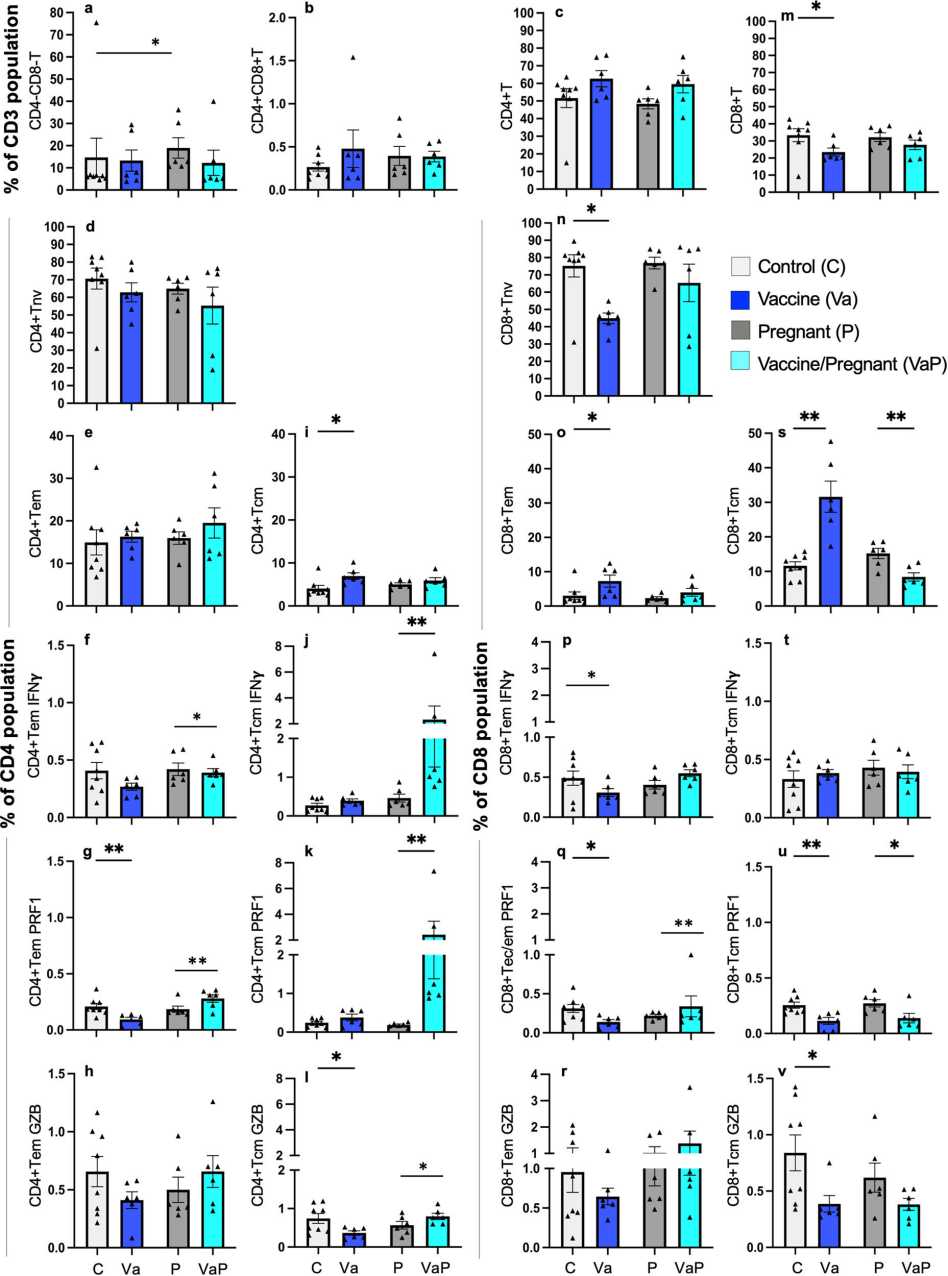
Ex vivo profiling of vaccine-induced T cells in pregnant mice. C57BL/6 female mice were immunized with nano2/4, mated with males at 28 days after 2nd vaccine dose, and euthanized at E12–17 gestation days. Non-mated, non-pregnant females were included as controls. Single-cell suspensions of splenic cells were labeled with fluorophore-conjugated antibodies as described in the “Methods”. CD3+ T cells based on the expression levels of CD4, CD8, CD62L, and CD44 were acquired by flow cytometry and analyzed by FlowJo software. Shown are percent frequencies of (**a**) CD4^−^CD8^−^, (**b**) CD4+CD8+T lymphocytes, and (**c**–**l**) CD4^+^ and (**m**–**v**) CD8^+^ T-cell subsets that exhibited naive (Tnv, d, n), effector/effector memory (Tem, **e**, **o**), and central memory (Tcm, **i**, **s**) phenotypes and produced IFNγ (**f**, **j**, **p**, **t**), Perforin (PRF1, **g**, **k**, **q**, **u**) or Granzyme B (GZB, **h**, **l**, **r**, **t**). Each mouse value is presented by a triangle and mean ± SEM values derived from duplicate observations per sample are plotted. Number of females per experiment: C (*n* = 8), Va (*n* = 6), P (*n* = 6), VaP (*n* = 6). Significance was calculated by Students’ unpaired *t*-test with or without Welch’s correction or Mann–Whitney *U*-test and *p*-values of <0.05 and <0.01 are annotated with one and two symbols, respectively. The horizontal bar indicates the compared groups. Detailed data are presented in Supplementary Table 2.

To examine if vaccine efficiency in eliciting *Tc-*specific T-cell response is altered during pregnancy, we analyzed the splenic cells ex vivo and after in vitro stimulation with antigenic *Tc* lysate (TcL) by flow cytometry. Information regarding antibodies used for flow cytometry is presented in Supplementary Table 1. The gating strategy for capturing the frequencies of T-cell subsets is shown in Supplementary Fig. 1. Data from ex vivo flow analysis are shown in Fig. 2 and Supplementary Table 2. Immunization with nano2/4 elicited no significant changes in the frequencies of double negative (DN, CD4–CD8–), double positive (DP, CD4+CD8+) and CD4+ total, naive (Tnv) and effector/effector memory (Tem) T-cell subpopulations in control and pregnant mice (Fig. 2a–e). Functional activation of CD4+Tem, examined by IFNγ, perforin (PRF1), and granzyme B (GZB) production, was not induced by nano2/4 in non-pregnant mice, while vaccinated/pregnant mice exhibited increased frequency of PRF1-expressing CD4+Tem subset (Fig. 2f–h). A 72% increase in CD4^+^ central memory (Tcm) subset without an associated functional response was noted in vaccinated (vs. control) mice (Fig. 2i–l). In pregnant mice, nano2/4 did not signal an overall increase in the CD4+Tcm subset but led to 402%, 1206%, and 39% increase in the frequencies of IFNγ, PRF1, and GZB-producing CD4+Tcm subsets (Fig. 2i–l, *p* < 0.05–0.01). A decline in CD8+ total and Tnv subpopulations was compensated by a 141% and 172% increase in the CD8+ Tem and Tcm subsets, respectively, in vaccinated (vs. control, *p* < 0.05–0.01) mice, while pregnant mice exhibited non-significant changes in these CD8+T-cell subsets after nano2/4 immunization (Fig. 2m–o, s). Nano2/4 had no or slightly lowering effect on the functional response of CD8+ T subsets in control and pregnant mice (Fig. 2p–r, t–v).

Recall response to vaccine-induced T cells was examined after in vitro incubation for 48 h with antigenic TcL, and data are shown in Supplementary Fig. 2 and Supplementary Table 3. Briefly, TcL stimulation led to an expansion of the CD4+Tem subset, and decline in DP T cells in vaccinated/non-pregnant mice, and an increase in the CD4+Tnv subset in vaccinated/pregnant mice (*p* < 0.05). Importantly, TcL elicited 116% and 1293% increase in the frequencies of IFNγ- and PRF1-producing CD4+Tem subsets, respectively, in vaccinated/pregnant mice (*p* < 0.05–0.01). CD4+Tcm cells responded to TcL with a substantial (48%–470%) increase in IFNγ, PRF1, and GZB production in both non-pregnant and pregnant groups (*p* < 0.05–0.001). Regarding CD8+ T cells, TcL led to a slight decline in CD8+ and CD8+Tem subsets in pregnant mice, and an expansion of CD8+Tnv subset in pregnant and vaccinated/pregnant mice. The frequencies of IFNγ-, PRF1-, and GZB-producing CD8+Tem and CD8+Tcm subsets were not changed or slightly decreased after in vitro incubation with TcL in all vaccinated groups.

Together, the results discussed above suggest that nano2/4 signaled the expansion of CD4+Tem, CD8+Tem, and CD8+Tcm subsets (ex vivo or after TcL stimulation) in non-pregnant mice. Pregnancy had an overall dampening effect on the proliferation of CD8+ Tem and Tcm subsets. However, CD4 + T cells in vaccinated/pregnant mice were functionally activated that is evidenced by an increase in IFNγ, PRF1, or GZB production by CD4+Tcm subset. Our observation of higher proportions of CD4+ Tcm in response to vaccine in pregnant mice is in alignment with the documented increase in CD4+Tcm frequency in pregnancy in humans^27^.

### T-cell immune profile to challenge infection in pregnant mice (±nano2/4)

**Fig. 3:**
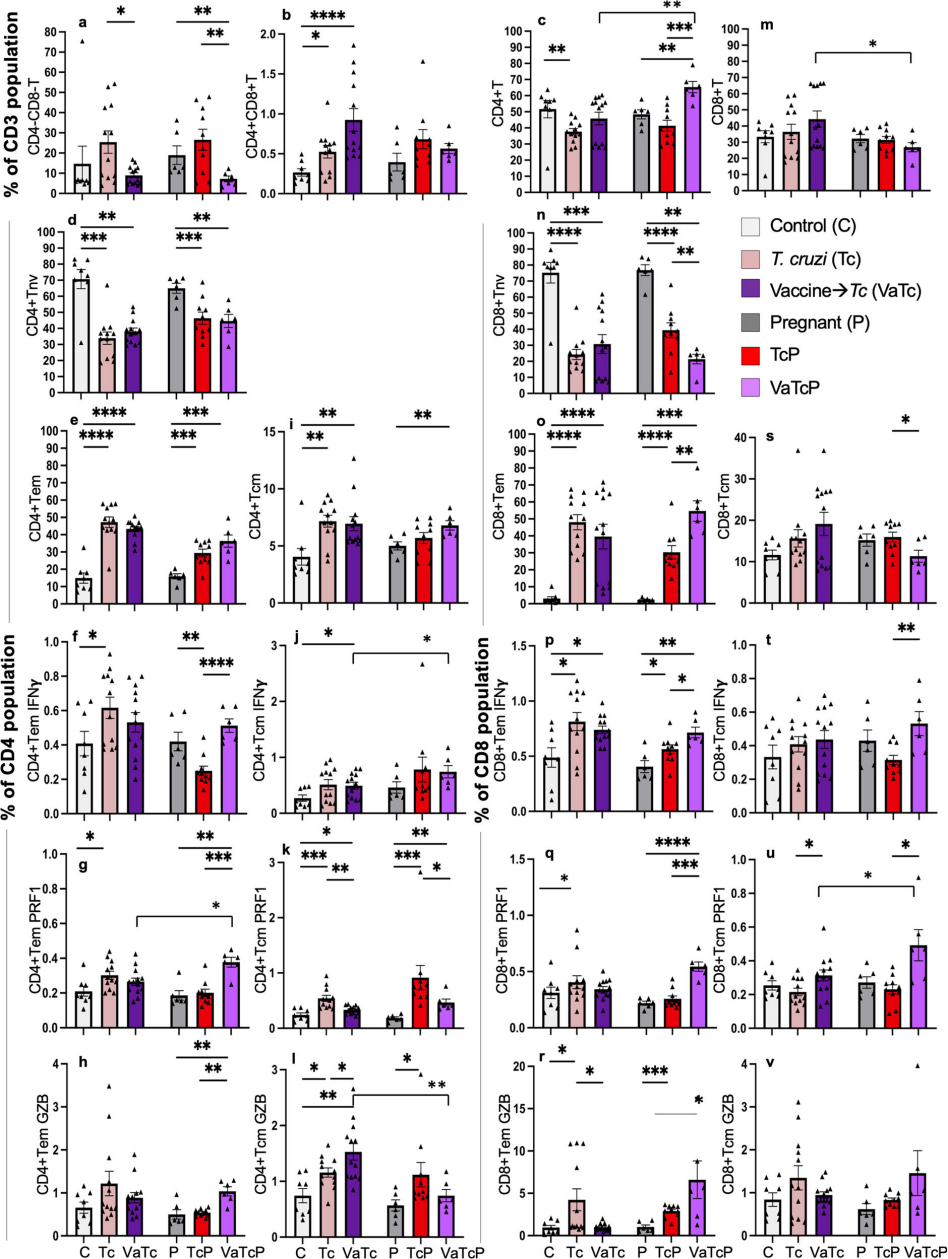
Expansion of vaccine-induced T cells after challenge infection in pregnant mice. C57BL/6 female mice were immunized with empty plasmid or nano2/4, challenged with *Tc* at 21 days post 2nd vaccine dose, mated with males at 7 days post-infection, and euthanized at E12–17 gestation days. Non-mated, non-pregnant female mice were included as controls. Splenocytes were labeled with fluorophore-conjugated antibodies and analyzed by flow cytometry, as in Fig. 2. Percent frequencies of **a** CD4^−^CD8^−^, **b** CD4+CD8+T lymphocytes, and (**c**–**l**) CD4^+^ and (**m**–**v**) CD8^+^ T-cell subsets that exhibited Tnv (**d**, **n**), Tem (**e**, **o**), and Tcm (**i**, **s**) phenotypes and produced IFNγ (**f**, **j**, **p**, **t**), PRF1 (**g**, **k**, **q**, **u**) and GZB (**h**, **l**, **r**, **v**) molecules are shown. Each mouse value is presented by a triangle and mean ± SEM values derived from duplicate observations per sample are plotted. Number of females: C (*n* = 8), *Tc* (*n* = 12), VaTc (*n* = 13), P (*n* = 6), TcP (*n* = 10), VaTcP (*n* = 6). Significance was calculated by Students’ unpaired *t-*test with or without Welch’s correction or Mann–Whitney *U*-test and *p-*values of <0.05, <0.01, <0.001, and <0.0001 are annotated with one, two, three, and four symbols, respectively. The horizontal bar indicates the compared groups. Detailed data are presented in Supplementary Table 4.

Next, we determined if host immunity to *Tc* infection is altered by pregnancy and if nano2/4 up-regulates the anti-parasite T-cell immunity during pregnancy (Fig. 3 and Supplementary Table 4). The expansion of DN T cells by challenge infection was eliminated by pre-immunization with nano2/4 while DP T cells continued to proliferate by 42%–150% in infected and vaccinated/infected non-pregnant groups (Fig. 3a, b). The contraction of CD4+Tnv cells (46%–52% decline) was followed by 190%–216% and 71%–77% expansion of CD4+Tem and CD4+Tcm subsets, respectively, in infected and vaccinated/infected non-pregnant mice (Fig. 3d, e, i, *p* < 0.01–-0.0001). In pregnant mice, challenge infection resulted in 29%–31% contraction of CD4+Tnv subset, and 84%–128% and up to 36% expansion of CD4+ Tem and Tcm subsets, respectively, the maximal response being noted in vaccinated/infected pregnant mice (Fig. 3d, e, i, *p* < 0.01–0.001). At a functional level, the frequencies of IFNγ- and PRF1-expressing CD4+Tem subsets were similarly increased (27%–51%) in infected and vaccinated/infected (vs. control) groups; while pregnant mice exhibited 22%–108% higher frequencies of IFNγ+, PRF1+, and GZB+CD4+Tem cells when they were vaccinated before challenge infection (Fig. 3f–h, *p* < 0.01–0.001). The CD4+Tcm cells producing IFNγ, PRF1, and GZB were increased by 38%–124% in infected and vaccinated/infected non-pregnant groups (Fig. 3j–l, *p* < 0.05–0.001). Functionally activated CD4+Tcm subsets were increased by 30.8%–393% after challenge infection in pregnant mice, with the higher extent of CD4+Tcm subsets stimulation being observed in infected (vs. vaccinated/infected) pregnant mice (Fig. 3j–l, *p* < 0.05–0.01).

Regarding CD8+ T cells, *Tc* infection resulted in 40%–72% decline in CD8+Tnv cells followed by 1187%–2242% expansion of CD8+Tem irrespective of pregnancy and vaccination status, and maximal increase in CD8+ Tem/Tnv ratio was observed in vaccinated/infected pregnant mice (Fig. 3m–o, *p* < 0.01–0.0001). Functionally active, IFNγ+CD8+Tem cells were increased by 52%–66% in infected and infected/vaccinated groups while PRF1+ and GZB+CD8+Tem subsets were increased by 32% and 343%, respectively, in infected mice only (Fig. 3p–r, *p* < 0.05–001, vs. control). In pregnant mice, IFNγ+ and GZB+CD8+Tem subsets were increased by 36.6% and 184% in response to challenge infection, and frequencies of these subsets were further increased by 29% and 127%, respectively, when pregnant mice were vaccinated before challenge infection (Fig. 3p, r, *p* < 0.05–0.001). The PRF+CD8+Tem subset was increased by 145% in vaccinated/infected (vs. infected-only or vehicle-only) pregnant mice (Fig. 3q, *p* < 0.001). The CD8+Tcm subset slightly contracted in vaccinated/pregnant mice in response to *Tc* infection (Fig. 3s), yet functional activation of CD8+Tcm, evidenced by 67%, 113%, and 76% increase in IFNγ, PRF1, and GZB-producing subsets, respectively, was primarily noted in vaccinated/infected (vs. infected) pregnant mice (Fig. 3t–v, *p* < 0.05).

We did not see robust further expansion of most of the T-cell subsets in vaccinated/infected (±pregnancy) mice after in vitro stimulation with TcL (see Supplementary Fig. 3 and Supplementary Table 5). At a functional level, TcL led to a 161% increase in GZB+CD4^+^Tem subset in vaccinated/infected mice (*p* < 0.05). A 96%–272% increase in IFNγ+, PRF1+, or GZB+ CD4^+^Tcm subsets was noted in all infected groups, and predominance of CD4+ T cells’ activation occurred in infected/vaccinated mice (*p* < 0.01–0.001). Except for IFNγ+ and PRF1 + CD4+Tem subsets that were increased by 31%–69% after TcL stimulation in infected/pregnant mice (*p* < 0.05), TcL did not enhance the functional activation of CD4 + T-cell subsets in infected/pregnant (±nano2/4) mice. Importantly, in vitro stimulation with TcL led to an 89–145% increase in GZB-producing CD8+Tem in infected (±nano2/4) mice (*p* < 0.01–0.0001) and a 15%–116% increase in IFNγ+ and GZB+CD8+Tem subsets in infected and infected/vaccinated pregnant mice (*p* < 0.05–0.001). Simultaneously, TcL led to a general decline in the frequencies of functionally activated CD8+Tcm subsets of infected and infected/vaccinated control and pregnant groups of mice.

Together, the results presented in Fig. 3 and Supplementary Fig. 3 suggest that (**a**) both control and pregnant mice respond to challenge infection with a substantial increase in differentiation, proliferation and functional activation of CD4+T and CD8+T subpopulations, (**b**) pre-immunization with nano2/4 elicited significantly higher level of expansion and functional activation of CD4+Tem and CD4+Tcm subsets in infected pregnant (but not non-pregnant) mice, and (**c**) increase in functional activation of CD8+Tcm subsets was primarily noted in vaccinated/infected pregnant mice. Because splenic cells have been antigenically activated twice with vaccine and once with challenge infection in vivo, (**d**) further in vitro stimulation with TcL resulted in insignificant expansion or contraction (likely due to cell death) of T-cell subsets, but it was effective in reactivating the functional CD4+Tcm subsets in non-pregnant mice, and functional CD8+Tem subsets in pregnant mice. Overall, these results show that nano2/4 expands the type 1 functional T-cell response against *Tc* infection in pregnancy.

### Systemic immune response in pregnant mice challenged with *Tc* (±nano2/4)

**Fig. 4:**
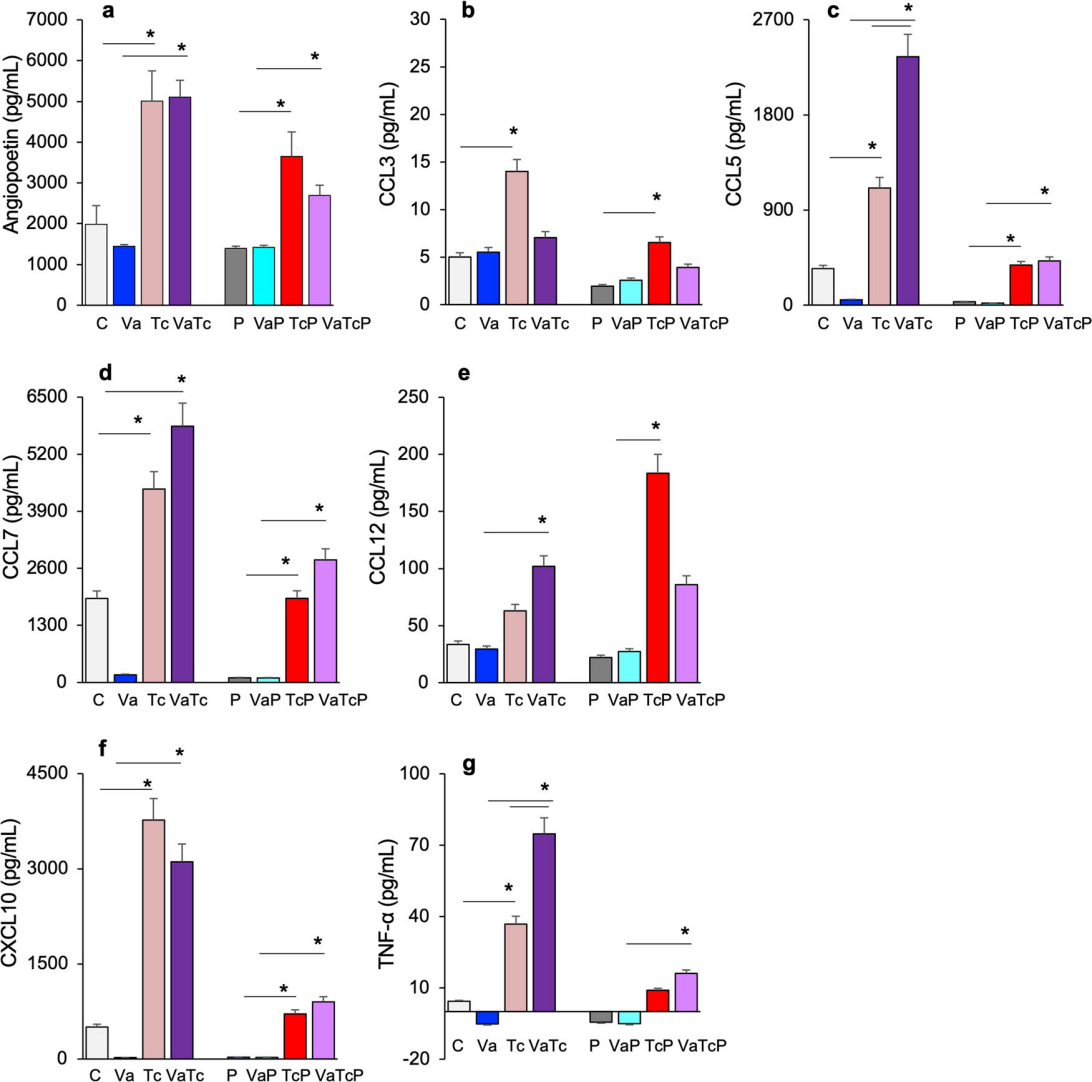
Systemic immune response in acutely infected pregnant mice (±nano2/4). Mice were immunized, infected, and mated, as described in Fig. 1a. Age-matched, similarly treated mice that were not mated were used as controls. Mice were euthanized at 19–24 days pi (= E12–17 gestation days in pregnant mice). Serum samples were analyzed in duplicate using a custom-designed Mouse Cytokine Luminex Performance 21-plex Panel. Shown are the angiopoetin (**a**), CCL3 (**b**), CCL5 (**c**), CCL7 (**d**), CCL12 (**e**), CXCL10 (**f**) and TNF alfa (**g**) levels that changed significantly in response to vaccination and/or challenge infection in pregnant and non-pregnant mice. Data are presented as mean ± SEM values (*n* = 5–10 per group). Significance was calculated by Students’ unpaired *t*-test with or without Welch’s correction or Mann–Whitney *U*-test and *p*-values of <0.05 are plotted. The horizontal bar indicates the compared groups.

We measured a panel of cytokines/chemokines in the serum of infected and infected/vaccinated groups of non-pregnant and pregnant mice to evaluate the systemic immune profile. For this, serum samples of all groups of mice were analyzed using a Multiplex Cytokine Assay. Of the 21 analytes that were evaluated, 9 analytes (angiopoietin, CCL3, CCL5, CCL7, CCL12, CXCL10, and TNF-α) were significantly changed in response to infection in both non-pregnant and pregnant mice, and more robust response was, in general, noted in non-pregnant mice (Fig. 4a–g, *p* < 0.05). Pre-immunization with nano2/4 resulted in a further increase in circulatory CCL5, CCL7, CCL12, and TNF-α levels in infected/vaccinated (vs. infected) mice, while infected/pregnant mice exhibited no further increase or a decline in the serum cytokines when they were pre-vaccinated with nano2/4 (Fig. 4a–g, *p* < 0.05). These results suggest that both pregnant and non-pregnant mice respond to *Tc* infection (±nano2/4) with the production of cytokines and chemokines, though pregnant mice tend to prevent the overwhelming damaging immune activation.

### Pathogenesis of Chagas disease in pregnant mice (±nano2/4)

**Fig. 5:**
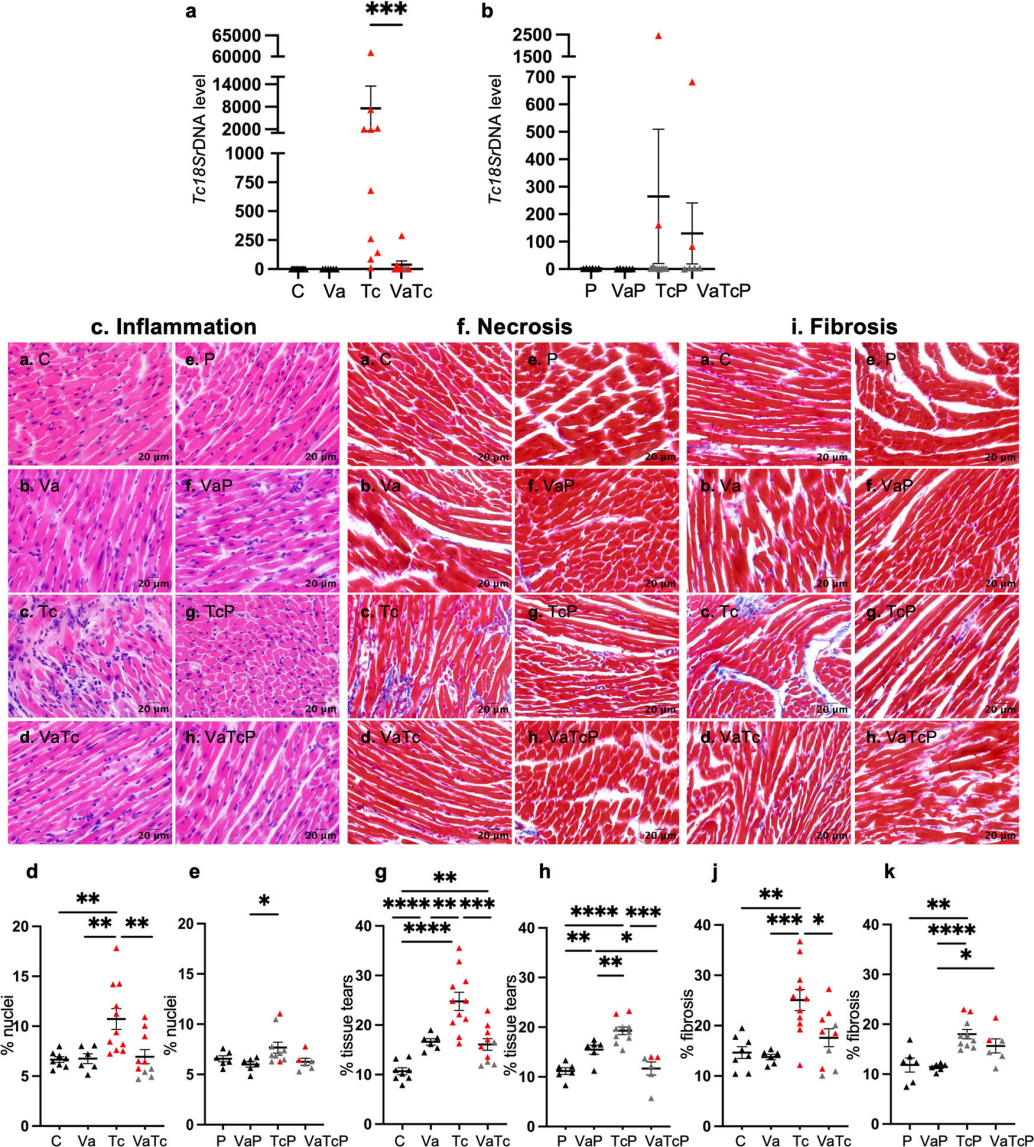
Vaccine efficacy in controlling *Tc*-induced cardiac pathogenesis in pregnant mice. Female mice were immunized with nano2/4, infected with *Tc*, and mated with males as described in Fig. 1a. Similarly treated but not mated females were used as controls. Parasite burden. Total DNA was isolated from heart tissues of control (**a**) and pregnant (**b**) groups and submitted to real-time qPCR amplification of *Tc*18SrDNA sequence (normalized to murine Gapdh). Parasite burden and percent of mice positive for *Tc* are plotted as a colored distinction between mice that were not infected (black triangle), and mice with detectable (red triangle) or undetectable *Tc* (gray triangle). Histology. Paraffin-embedded 5-µm heart tissue sections were subjected to (**c**, **f**) H&E and (**i**) Masson’s Trichrome staining, and representative images identifying inflammatory infiltrate, necrosis, and fibrosis in control (panels **a**–**d**) and pregnant (panels **e**–**h**) female groups are shown (scale bar: 20 μm). Thresholding methods with Image J software were applied to calculate the percent nuclei as a measure of inflammatory infiltrate (**d**, **e**), tissue tear as a measure of necrosis (**g**, **h**), and blue-colored collagen deposition as a measure of fibrosis (**j**, **k**), as described in methods. Number of females: C (*n* = 8), Va (*n* = 6), Tc (*n* = 11), VaTc (*n* = 10), P (*n* = 6), VaP (*n* = 6), TcP (*n* = 10), VaTcP (*n* = 6). In graphs, each mouse value is presented by a triangle, and mean ± SEM values derived from 2 tissue sections per mouse (each slide analyzed in >9 microscopic fields) are plotted. Significance was calculated by Students’ unpaired *t*-test with or without Welch’s correction or Mann–Whitney *U*-test and *p-*values of <0.05, <0.01, <0.001, <0.0001 are annotated with one, two, three, and four symbols, respectively. The horizontal bar indicates the compared groups. Detailed data are presented in Supplementary Table 6.

**Fig. 6:**
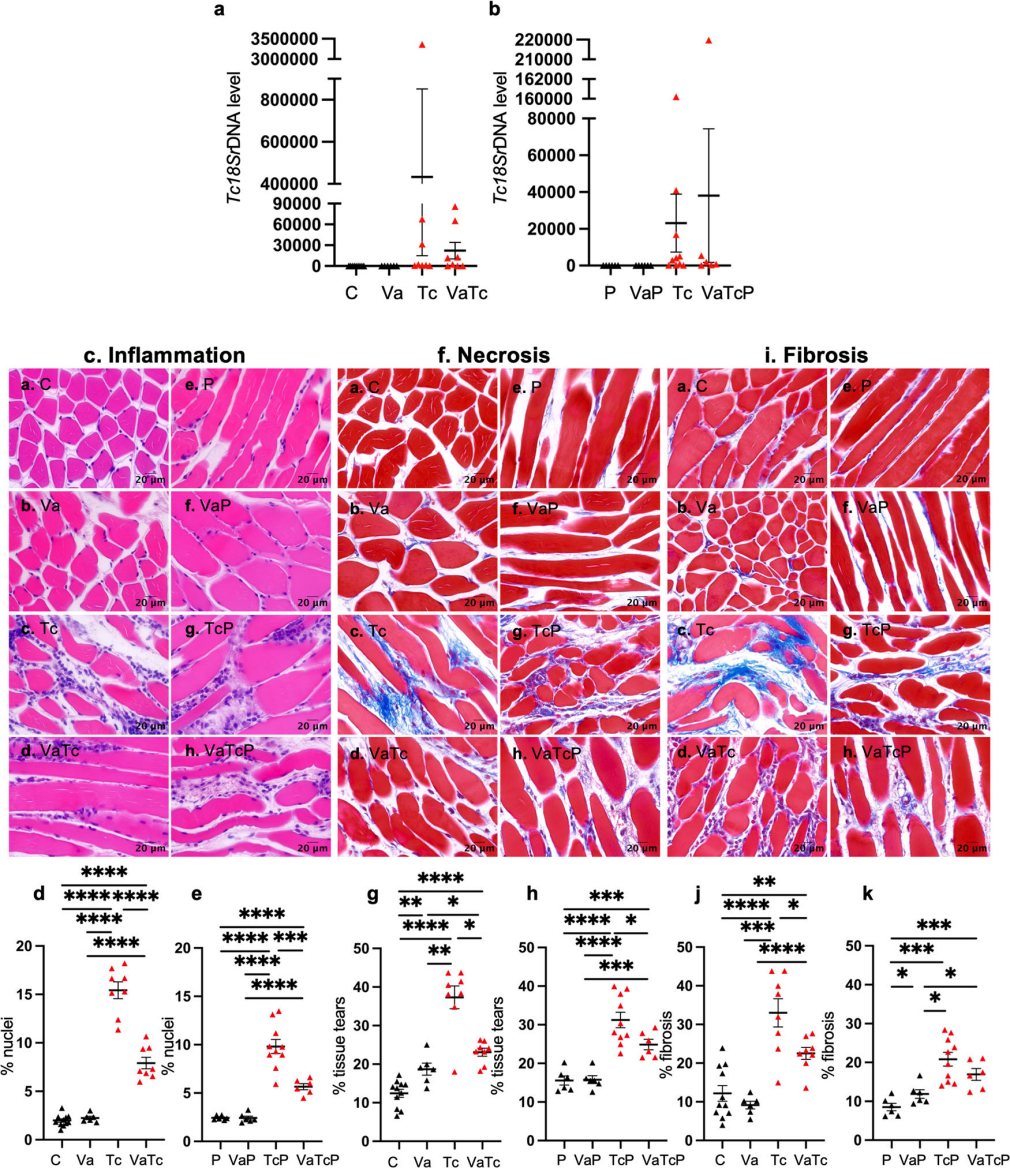
Vaccine efficacy in controlling *Tc*-induced inflammatory infiltrate, necrosis, and fibrosis in the skeletal muscle of pregnant mice. Female mice were immunized with nano2/4, infected with *Tc*, and mated with males. Similarly treated but not mated females were used as controls. Parasite burden. Total DNA isolated from SkM tissues of control (**a**) and pregnant (**b**) groups was analyzed by real-time qPCR for *Tc*18SrDNA sequence (normalized to murine Gapdh). Parasite burden and percent of mice positive for *Tc* are plotted as a colored distinction between mice that were not infected (black triangle) and mice with detectable (red triangle) or undetectable *Tc* (gray triangle). Histology. Paraffin-embedded 5-µm SkM tissue sections were subjected to (**c**, **f**) H&E and **i** Masson’s Trichrome staining and representative images identifying inflammatory infiltrate, necrosis and fibrosis in control (panels **a**–**d**) and pregnant (panels **e**–**h**) female groups are shown (scale bar: 20 μm). Thresholding methods with ImageJ software were applied to calculate the percent nuclei as a measure of inflammatory infiltrate (**d**, **e**), tissue tear as a measure of necrosis (**g**, **h**), and blue-colored collagen deposition as a measure of fibrosis (**j**, **k**), as described in methods. Number of females: C (*n* = 11), Va (*n* = 6), Tc (*n* = 8), VaTc (*n* = 8), P (*n* = 6), VaP (*n* = 6), TcP (*n* = 10), VaTcP (*n* = 6). In graphs, each mouse value is presented by a triangle, and mean ± SEM values derived from two tissue sections per mouse (each slide analyzed in >9 microscopic fields) are plotted with a horizontal line. Significance was calculated by Students’ unpaired *t*-test with or without Welch’s correction or Mann–Whitney *U*-test and *p*-values of <0.05, <0.01, <0.001, <0.0001 are annotated with one, two, three, and four symbols, respectively. The horizontal bar indicates the compared groups. Detailed data are presented in Supplementary Table 6.

We analyzed the tissue parasite burden by qPCR and tissue inflammatory infiltrate, necrosis, and fibrosis by histology to determine if the vaccine provided protection from tissue infection and damage during pregnancy (Figs. 5 and 6; Supplementary Table 6). After challenge infection, cardiac parasite burden was detectable in 100% of the non-pregnant mice and 40% of pregnant mice, and it occurred at >10-fold higher level in non-pregnant (vs. pregnant) mice despite the similar level of initial infection dose (Fig. 5a, b). Thus, a higher efficacy of the vaccine in decreasing the parasite burden by 99% was observed in the infected, non-pregnant group (*p* < 0.001), while this effect was not as evident in infected/pregnant mice (Fig. 5a, b). Histological staining with H&E identified no cardiac inflammation in control and pregnant mice before and after vaccination (Fig. 5c–e). Challenge infection induced a 61% and 17% increase in cardiac inflammatory infiltrate in non-pregnant and pregnant mice, respectively, and it was completely controlled upon pre-immunization with nano2/4 (Fig. 5c–e, *p* < 0.05–0.01). The vaccine alone caused mild myocardial tissue damage, and it was increased by 56% and 38% after challenge infection in control and pregnant mice, respectively (Fig. 5f–h). Immunization with nano2/4 abolished the *Tc*-induced cardiac tissue damage in pregnant mice, while it was partially controlled in non-pregnant mice (Fig. 5f–h, *p* < 0.05–0.0001). Histological staining with Masson’s Trichrome identified no cardiac fibrosis in control and pregnant mice before and after vaccination (Fig. 5i–k). *T. cruzi* challenge induced a 71% and 52% increase in cardiac fibrosis in non-pregnant and pregnant mice, respectively, and it was partially controlled upon pre-immunization with nano2/4 (Fig. 5i–k, *p* < 0.05–0.001).

A similar analysis of parasite burden and tissue damage was performed in the skeletal muscle of all mice (Fig. 6). Because of the myotropic nature of the parasite, *Tc* was detected in SkM of all mice after challenge infection, albeit it was detected at >15-fold higher levels in non-pregnant mice than the pregnant mice (Fig. 6a, b). A higher efficacy of the vaccine in decreasing the parasite burden by 95% was observable in the infected control group, while this effect was not evident in infected/pregnant mice (Fig. 6a, b). Mild tissue necrosis but no inflammation and fibrosis were noted in the skeletal muscle of control and pregnant mice before and after vaccination (Fig. 6c–k). Challenge infection induced 674% and 309% increase in inflammatory infiltrate, 181% and 71% increase in tissue necrosis, and 171% and 145% increase in fibrosis in skeletal muscle of non-pregnant and pregnant mice, respectively (Fig. 6c–k, *p* < 0.01–0.001). Importantly, immunization with nano2/4 led to significant control of *Tc-*induced SkM inflammation, necrosis, and fibrosis in both non-pregnant and pregnant groups (Fig. 6c–k, *p* < 0.05–0.001).

Together, the results presented in Figs. 5 and 6 show that (a) tissue inflammatory infiltrate, necrosis, and fibrosis were significantly arrested by pre-immunization with nano2/4 in both pregnant and non-pregnant mice, and (b) pregnant mice exhibited more pronounced control of tissue damage post-vaccination. Further, (c) nano2/4 offered >95% control of parasite burden in muscle tissues of non-pregnant mice. The finding of lower parasite burden in the heart and skeletal tissues of pregnant mice could potentially be explained by the hiding of the parasite in other tissues, such as adipose tissue or gastrointestinal tissues, which are known reservoirs for the parasite, or by the effects of the pregnancy-related hormonal changes. This hypothesis remains to be tested in future studies.

### Vaccine efficacy in modulating the placental outcomes in pregnant mice (±*Tc*)

**Fig. 7:**
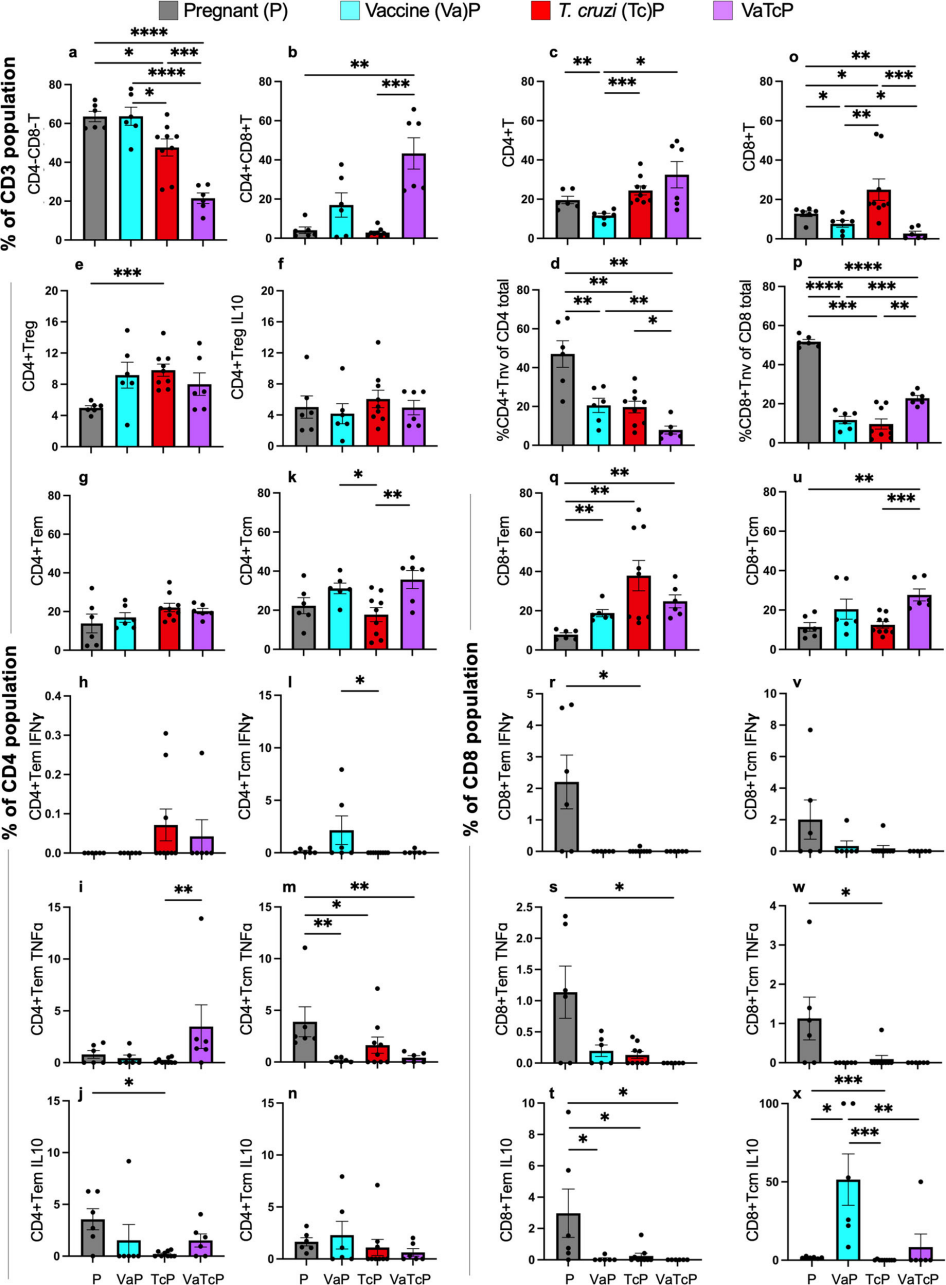
Placental immune response to nano2/4 and *Tc* infection in pregnant mice. C57BL/6 female mice were immunized with nano2/4, infected with *Tc* at 21 days after 2nd vaccine dose, mated with males at 7 days pi, and euthanized at E12–17 gestation days. Single-cell suspensions of placental cells from pregnant mice that were non-infected (P), vaccinated (VaP), *Tc-*infected (TcP), or vaccinated/infected (VaTcP) were labeled with fluorophore-conjugated antibodies and analyzed by flow cytometry equipped with FlowJo software. Bar graphs show the percent frequencies of **a** CD4^−^CD8^−^ and **b** CD4^+^CD8^+^ T lymphocytes, and **c**–**n** CD4+ and **o**–**x** CD8+ T-cell subsets that exhibited Treg (**e**), Tnv (**d** & **p**), Tem (**g** & **q**), or Tcm (**k** & **u**) phenotypes and produced IFNγ (**h**, **l**, **r**, **v**), TNFα (**i**, **m**, **s**, **w**) or IL10 (**j**, **n**, **t**, **x**) cytokines. Number of females: P (*n* = 6), VaP (*n* = 6), TcP (*n* = 9), VaTcP (*n* = 6). Data from each dam is presented by a triangle and mean ± SEM values derived from duplicate observations per placental sample are plotted. Significance was calculated by Students’ unpaired *t*-test with or without Welch’s correction or Mann–Whitney *U*-test and *p-*values of <0.05, <0.01, <0.01, and <0.0001 are annotated with one, two, three, and four symbols, respectively. The horizontal bar indicates the compared groups. Detailed data are presented in Supplementary Table 7.

Finally, we focused on determining if the benefits of maternal nano2/4 immunization are transferred to the developing fetal tissues that otherwise are exposed to maternal *Tc* infection-induced pathological sequelae. For this, we first examined the placental immune profile by flow cytometry in the pregnant groups (Fig. 7 and Supplementary Table 7) using the gating strategy shown in Supplementary Fig. 1. The vaccine alone elicited placental contraction of CD4+ and CD8+ (total and naive) subsets and an increase in CD8+Tem and IL10+CD8+Tcm subsets (Fig. 7c, d, o–q, x, *p* < 0.05–0.01). Challenge infection resulted in a significant increase in placental frequencies of CD8+ (total and Tem) subsets in pregnant mice (Fig. 7o, q, *p* < 0.01). Immunization with nano2/4 before infection led to a pronounced decline in DN, CD4+Tnv and significantly higher expansion of DP, CD4+ (total, Tcm) and CD8+ (Tnv, Tcm) subsets than was observed in placentas of infected-only mice (Fig. 7). Specifically, TNFα+CD4+Tem subset was increased by 338% (*p* < 0.01) in placentas of vaccinated/infected mice (Fig. 7i). Placental CD4+Treg cells were increased by 61%–97% (Fig. 7e); however, a functional activation of Treg cells in response to vaccination and/or infection was not noted.

**Fig. 8:**
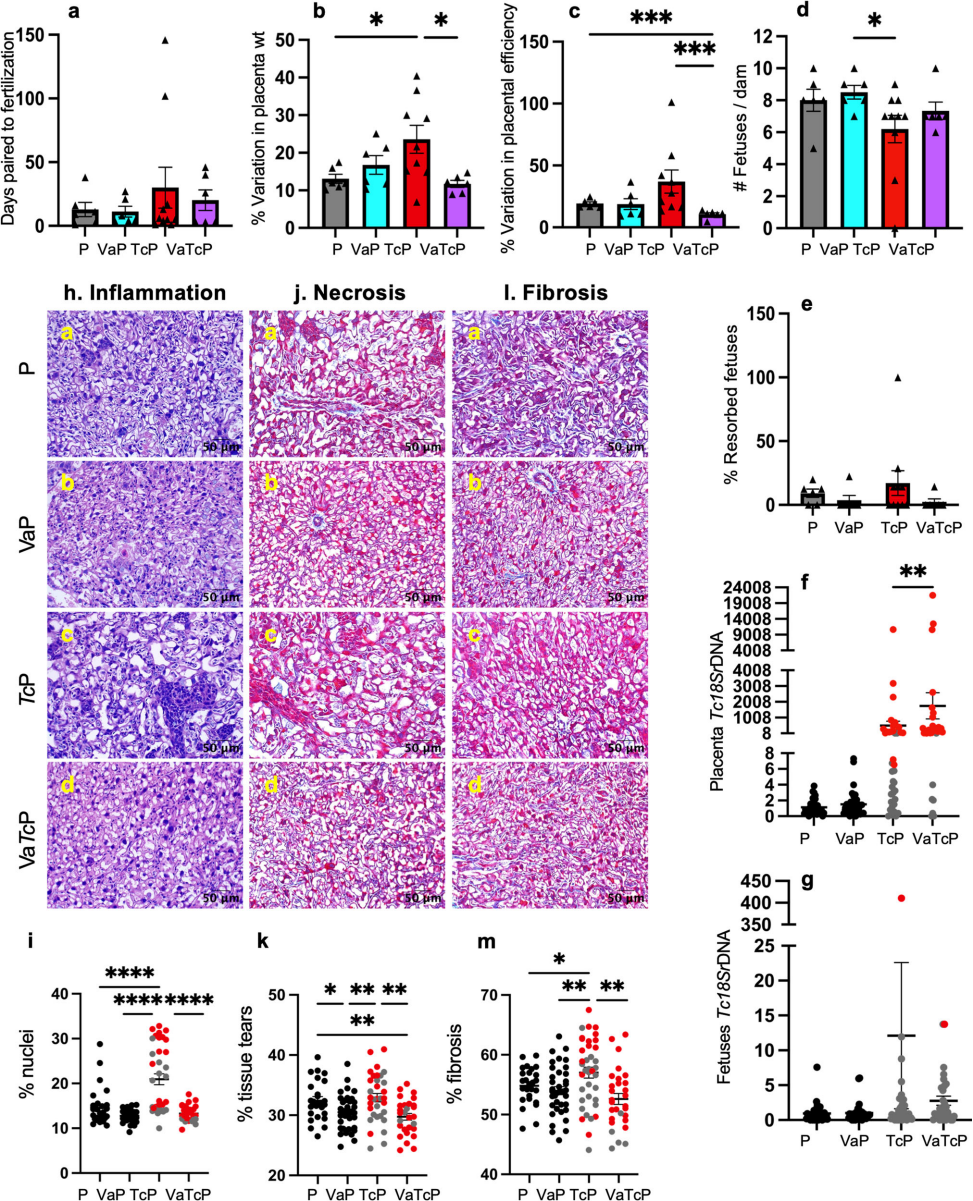
Vaccine efficacy in improving the placental/fetal outcomes in infected pregnant mice. C57BL/6 female mice were prophylactically immunized with nano2/4, infected with *Tc* at 21 days post 2nd vaccine dose, mated with males at 7 days post-infection, and euthanized at 12–17 gestation days. Pregnancy outcomes. Breeding time post-pairing (**a**), percent variation in placental weight (**b**), placental efficiency (**c**), and frequencies of full-term (**d**) and resorbed (**e**) fetuses were recorded. Parasite burden. Placental (**f**) and fetal (**g**) parasite burden were evaluated by real-time qPCR measurement of *Tc*18SrDNA sequence (normalized to murine Gapdh). Histology. Paraffin-embedded 5-µm placental tissue sections were stained with H&E (**h**, **j**) and Masson’s Trichrome (**l**). Representative placental images of untreated/uninfected pregnant mice (P) and pregnant mice that were vaccinated (VaP), infected (TcP), or vaccinated/infected (VaTcP) are shown (scale bar: 50 μm). Thresholding methods with ImageJ software were applied to calculate the percent nuclei as a measure of inflammatory infiltrate (**i**), tissue tear as a measure of necrosis (**k**), and blue-colored collagen deposition as a measure of fibrosis (**m**). At least two tissue sections per sample were scored in 9–10 microscopic fields. In graphs, each sample value is presented with a triangle, and mean values ± SEM values are plotted. Sample size for **a**–**e**: P (*n* = 6), VaP (*n* = 6), TcP (*n* = 10), VaTcP (*n* = 6). The sample size for **f**–**m**: (females/placentas or fetuses): P (*n* = 6/28-32), VaP (*n* = 6/37), TcP (*n* = 10/36–39), VaTcP (*n* = 6/30–31). Significance was calculated by Students’ unpaired *t*-test (U) with or without Welch’s correction (W) or Mann–Whitney *U*-test (MW) and *p*-values of <0.05, <0.01, <0.01, and <0.0001 are annotated with one, two, three, and four symbols, respectively. The horizontal bar indicates the compared groups. Detailed data are presented in Supplementary Table 8.

Vaccine-induced placental immunity was associated with improved pregnancy outcomes (Fig. 8 and Supplementary Table 8). This was evidenced by the findings that female mice infected with *Tc* (vs. non-infected or vaccinated-only) exhibited a delay in fertilization (134%–169% increase), and variance in placental weight (indicates a percent coefficient of variation for the parameters determines that the higher the percent, the greater the dispersion around the mean between the dams, 41%–80% increase, *p* < 0.05) and placental efficiency (indicates greater variability among fetal to placenta ratio and the inability to carry all fetuses to maturity, 92%–97% increase, *p* < 0.05) along with >20% decline in the number of mature fetuses (*p* < 0.05) and a substantial increase in resorbed fetuses (Fig. 8a–e). The vaccine had no adverse effects on the pregnancy outcomes, and immunization with nano2/4 before challenge infection prevented the fetal loss and normalized the fertilization time, placental weight, placental efficiency, and number of mature fetuses to the levels noted in non-infected and vaccinated-only females (Fig. 8a–e). Placental parasites were noted in 78% of the infected (±nano2/4) females, and it was not decreased in frequency but rather slightly increased in burden by vaccination. Maternal parasite transmission did not occur in >90% of the developing fetuses, irrespective of vaccination status. Representative images of placental tissues from all groups of pregnant mice documenting inflammatory infiltrate (Fig. 8h), necrosis (Fig. 8j), and fibrosis (Fig. 8l) are shown. Semi-quantitative evaluation of these parameters showed that *Tc* infection resulted in a 44% increase in placental inflammatory infiltrate, and immunization with nano2/4 was effective in preventing the *Tc-*induced placental inflammation (*p* < 0.0001) (Fig. 8i). Extensive necrosis (tissue tear) and fibrosis were not noted in placental tissues of the studied groups.

Together, the results presented in Figs. 7 and 8 suggest that nano2/4 DNA vaccine can modify the placental T-cell activation characteristics and functional phenotypes during pregnancy and *Tc* infection. While the placenta itself provided significant protection to developing fetus from maternal *Tc* infection, vaccination prevented the *Tc*-induced adverse effects on the placental function and fetal outcomes that was evidenced by normalization of fertilization time, placental weight, placental efficiency, and number of mature fetuses with no detection of fetal resorption to the levels noted in non-infected and vaccinated-only females. *Tc* induced placental inflammation was also controlled by pre-vaccination.

## Discussion

Our previous studies documenting the design of a DNA vaccine consisting of pCDNA3 encoding TcG2 and TcG4 candidates that provided protection from *Trypanosoma cruzi* infection in mice and dogs have provided the proof-of-principle for further development of a vaccine against CD^14–18^. Because of regulatory concerns, such as the potential integration of antibiotic-selective markers into the chromosome, pCDNA3 is not suitable for use in humans. Therefore, we subcloned the TcG2 and TcG4 vaccine candidates into a nanoplasmid designed under FDA regulatory guidelines^20^. Within the scope of this investigation, an experimental mouse model was used to evaluate the prophylactic efficacy of nano2/4 against *Tc* infection during pregnancy. We undertook this study because of several reasons. One, approximately 2-million women of childbearing age are infected, leading to over 18,000 infected infants being born each year^6,28^. Children exposed to *Tc* in early life may be susceptible to other infectious diseases^9^; and at higher risk of myocarditis, meningoencephalitis, and neurological behavioral and cognitive impairments as young adults^1,8^. Anti-parasitic drugs, benznidazole, and nifurtimox, are not recommended for the treatment of pregnant women because of their potential adverse effects on the developing fetus^10^. Moreover, *Tc* isolates from heterogenous backgrounds do not exhibit equal susceptibility to these drugs^29^. Thus, *Tc* infection presents an underappreciated health risk, highlighting the potential for adverse outcomes in proceeding generations. Second, an immunoregulatory environment during pregnancy is believed to be essential for the growth and health of the fetus, yet inactivated influenza, Tdap, and COVID vaccines have proven to be immunogenic and offer substantial benefits against infections and disease for both mothers and infants^30^. Third, pathogen-based attenuated vaccines are shown to confer protection against smallpox, plague, and measles^31^, yet, previous findings from our laboratory indicate that primary *Tc* infection induces delayed and suboptimal T-cell immunity, which fails to provide an effective defense against re-infection^32^. Thus, vaccines composed of attenuated parasites are unlikely to offer protection against *Tc*, also confirmed in several studies^33^. A subunit vaccine has been recommended to be the best choice for controlling *Tc* infection.

Herein, we evaluated the protective efficacy of the nano2/4 against maternal *Tc* infection and determined if the vaccine improves the pregnancy outcomes in infected female mice. We noted no physical, clinical, or inflammatory toxicity of the vaccine in pregnant and non-pregnant mice. Nano2/4 signaled the protective immunity in pregnancy that was evidenced by (a) expansion of CD4+Tem and CD4+Tcm subsets after immunization, (b) functional activation of CD4+T-cell subsets upon in vitro stimulation, and (c) increased proliferation of CD4+Tem, CD4+Tcm and CD8+Tcm subsets producing IFNγ and cytolytic molecules (PRF1, GZB) after challenge infection. Pre-vaccination decreased the inflammatory infiltrate, necrosis, and fibrosis in the myocardium and skeletal muscle of infected/pregnant mice. Nano2/4 also enhanced the anti-parasite antibody response and elicited a balanced serum cytokines/chemokines response along with placental T-cell immune characteristics that arrested the *Tc*-induced placental inflammation and improved the placental function and fetal outcomes in pregnant mice. This, to the best of our knowledge, is the first study testing the efficacy of a subunit vaccine in controlling the *Tc*-associated pathologies and improving the pregnancy outcomes in any CD model.

During embryogenesis, the placenta provides growth factors and hormones and serves as a physical and immune barrier against infectious agents for the developing fetus. Humans and mice share the discoid choriovillous shape of the placenta and the hemochorial composition of the maternal-fetal structure^34^. In humans, the placenta is characterized by a hemomonochorial structure (i.e., a single layer of syncytiotrophoblast), while in mice, it is hemotrichorial (i.e., one layer of cytotrophoblasts with two layers of syncytiotrophoblasts)^35^. However, in both cases, the hemochorial placenta enables direct contact between fetal trophoblast cells and maternal blood^34^. The notable difference in placental villous structure between mice and humans is related to maternal-fetal exchange and endocrine functions^36^. Among the various similarities and differences in anatomy and histology between human and mouse placentas, mice offer a valuable model for comprehending the impact of various stimuli on pregnancy outcomes. The large litter size, short gestational period, and the availability of immunological reagents also make mice a biologically relevant model for studying the impact of maternal infections and treatments on fetal survival and growth.

During immune activation, antigen presentation through the engagement of major histocompatibility complex (MHC) I and MHC II signals CD8^+^ and CD4^+^ T-cell activation, respectively. CD4^+^ cells can differentiate into various T helper subsets (e.g., Th1, Th2, Th17) or T regulatory cells to shape the pathological, protective, or regulatory immunity through the secretion of a diverse set of cytokines. Following the primary immune response, a subset of CD4^+^T cells differentiates into memory T cells. Similarly, CD8^+^ T cells differentiate as mature effectors that release cytotoxic molecules and cytokines for pathogen control and as central memory cells to provide protection against future infections^37^. Our current findings show that nano2/4 primed the functional activation of splenic effector and memory CD4+ T-cell subsets in pregnant mice and these cells were capable of producing parasite-killing molecules when encountering the antigenic stimulation. Indeed, challenge infection with *Tc* led to significant expansion of effector CD4+ and CD8+ T cells and production of cytotoxic molecules (IFNγ, PRF1, GZB) by both CD4+ and CD8+ effector T-cell subsets in vaccinated (vs. non-vaccinated) pregnant mice. However, we did not observe a significant increase in the frequencies of central memory T-cell subsets in infected or vaccinated/infected pregnant mice, likely because all mice were still experiencing the acute parasitemia phase and the primary effector response had not subsided. Nevertheless, the effector T-cell response was sufficient to provide protection from maternal infection and tissue pathogenesis. Systemic increase in the proinflammatory cytokines/chemokines by nano2/4 also correlated with control of parasites in maternal tissues. No studies have previously analyzed the T-cell phenotypic and functional response in *Tc*-infected pregnant mice treated with any vaccine. Others have noted that DNA vaccine-induced splenic CD8+ T-cell response and IFNγ production prevented the Zika viral infection and fetal damage in mice^38^. IFNγ production by NK and CD4^+^ T cells has also been attributed to control *T. gondii* in mice^39^. Future studies are needed to determine if the vaccine-induced maternal T-cell immunity, along with the antibodies provide protection to the neonates from future exposure to *Tc*.

Sterile immunity is rarely (if ever) seen against *Tc*, and it is generally accepted that the purpose of a vaccine should be to bring the parasite persistence below a threshold level that will prevent chronic tissue damage and symptomatic CD. In general, qPCR is considered the most sensitive and specific method for obtaining a quantitative measure of the changes in parasite burden^40,41^. *Tc* SylvioX10 isolate used in this study exhibits myotropic behavior and our findings show that nano2/4 vaccine provided up to 99% control of parasite burden in the myocardium and skeletal muscle of both pregnant and non-pregnant mice. Patients and mice with Chagas disease exhibit myocardial inflammatory infiltrate and profibrotic response^42^ that result in structural changes, increased stiffness, and abnormal left ventricular function, ultimately leading to cardiac failure^43^. It is worth noting that nano2/4 immunization led to decreased infiltration of immune cells, necrosis, and fibrosis in the cardiac and skeletal muscle tissues of pregnant and non-pregnant mice than was noted in non-vaccinated/infected mice. These findings show that nano2/4 was successful in diminishing the *Tc* infection and its pathogenic effects on host tissues during pregnancy. More studies are needed to determine if the vaccine can alleviate congenital transmission of the parasite to newborns.

CD4^+^ T cells constitute 30–45% of the immune cells at the maternal-fetal interface^44^, and 5% of these are CD4+ T regulatory (Treg) cells, which play a crucial role in facilitating placentation and fetal tolerance. CD8+ T cells are present in small quantities in the decidua, and exhibit lower expression of cytotoxic molecules (e.g., granzyme, perforin) than the CD8 + T cells in circulation^45^. TNFα exerts harmful effects on the placenta^46^ and IFNγ may serve a dual purpose by promoting the differentiation of decidual natural killer cells and facilitating the formation of the placenta in mice^47^. IL10 (immunoregulatory cytokine) deficiency can intensify pathological inflammation in various infections^48^, yet, pregnant IL10^−/−^ mice exhibit no significant alterations in litter size or development than the wild-type mice, suggesting that IL10 is not essential for pregnancy^49^. In the context of *Tc* infection, increased gene expression of innate immune responses and IFNγ and IL10 cytokines was noted in human placental explants exposed to *Tc*^50^. Others have shown that human *Tc*-induced placenta villi inflammation predominantly consisted of CD8^+^ T cells^51^. Our data showing that the nano2/4 vaccine influences the placental T-cell activation characteristics is likely the first study where immunological impact on the placenta induced by the vaccine during *Tc* infection is determined. Current findings also demonstrate that maternal immunization with nano2/4 decreased the placental inflammation, necrosis, and fibrosis caused by *Tc* infection and improved the pregnancy outcomes evidenced by improved fertility and maturation of fetuses and decreased fetal resorption.

We did not study the effects of maternal *Tc* infection and vaccination on the fetal immune responses in this study, yet fetuses mount their own immune response to the transplacental export of *Tc* antigens. For example, Alarcon et al. noted the presence of inflammatory infiltrate and *Tc* antigens in the skeletal muscle of fetuses and IFNγ + CD4 + T cells along with deposits of IFNγ and IL10 in placenta, heart, and skeletal muscle of fetuses of the infected NMRI mice^52^. Congenital *Tc* infection triggered innate and adaptive immune responses in human fetuses^53,54^. Others indicated that transmission of *Tc* correlated with decreased IFNγ and CD4^+^ and CD8^+^ T cells in pregnant women^54^ and in utero exposure to maternal *Tc*, without maternal-fetal transmission, signals higher innate and adaptive immune responses in the developing fetuses^55–57^. Future studies evaluating the T-cell development and function in fetuses and offspring will be needed to gain mechanistic insight into the effects of maternal Tc infection and vaccination on the generation of protective immunity in newborns.

## Conclusions

This study provides important evidence in favor of using a vaccine to prevent maternal *Tc* infection and its adverse effects on pregnancy outcomes and fetal development. We demonstrate that nano2/4 is not toxic and equally immunogenic in pregnant mice as it was in non-pregnant mice. Further, nano2/4-induced parasite-specific, protective antibody response and CD4+ and CD8+ effector and memory T-cell subsets that were capable of proliferation with functional activation required to control the *T. cruzi* infection. A balanced serum cytokines/chemokines response and placental immune characteristics prevented the pathological effects of cytotoxic immune response in pregnancy. Importantly, nano2/4 was effective in arresting the *Tc*-induced tissue inflammatory infiltrate, necrosis, and fibrosis in maternal and placental tissues and improving maternal fertility, placental efficiency, and fetal survival in pregnant mice. Utilizing this mouse model presents a promising approach for longitudinally monitoring the health outcomes of newborns exposed to maternal *Tc* infection and evaluating the effectiveness of vaccines and therapies against congenital Chagas disease.

Some limitations of this study are noted. The Sylvio X10/4 strain of *T. cruzi* is a myotropic isolate that does not induce lethal phenotype C57BBL/6 mice^58,59^. In this first study, we used this model to ensure that parasite dissemination would occur, but maternal or fetal loss would be controlled. Additional studies will be needed to verify if the vaccine offers protective immunity against more virulent parasite isolates in different hosts during pregnancy. Further, measurement of circulating parasites at different time points by PCR and/or hemoculture needs to be conducted to determine if the vaccine offers long-term sterile immunity against maternal infection and/or congenital transmission.

## Materials and methods

### Ethics statement

All animal experiments were performed using the National Institutes of Health guidelines for the care and housing of laboratory animals and with protocols approved by the Institutional Animal Care and Use Committee (PHS Assurance # D16-00202 (A3314-01), Protocol number 805029) at the University of Texas Medical Branch, Galveston. All experiments were conducted in a biosafety level 2 approved laboratory and all personnel received appropriate ABSL2/BSL2 training.

### Parasites

*Tc* SylvioX10 isolate was purchased from ATCC (Manassas, VA). Mouse myoblast C2C12 cells (ATCC CRL-1772) were seeded in T75 flasks containing 10 mL of RPMI 1640 with L-glutamine supplemented with 10% fetal bovine serum (FBS), 1% penicillin-streptomycin, and 1% sodium pyruvate (all media components from ThermoFisher Scientific, Waltham, MA), and cultured at 37 °C, 5% CO_2_. At 70% confluence, cells were infected with *Tc* (1:3, cell: parasite ratio). Up to 50% of the medium was changed on alternate days. *Tc* replicates as an intracellular amastigote and differentiates from infective trypomastigotes that are released in the medium by cell lysis. Medium-containing trypomastigotes were collected in 15 mL conical tubes and sequentially centrifuged at room temperature at 290 rcf (g force) for 2 min to remove cell debris and at 3220 rcf for 8 min to pellet the parasites. Parasites were resuspended in 500 μL of PBS, and viable, parasites (moving, thin, free flagellum) were counted using a hemocytometer.

### Vaccine

GenBank contains the *T. cruzi* (CL/Brenner) sequences for cDNAs for TcG2 and TcG4 (AY727915 and AY727917, respectively)^12^. The full-length CL/Brenner cDNAs for the two antigens were cloned into pCDNA3.1 eukaryotic expression plasmid and recombinant plasmids were purified by anion exchange chromatography using a Qiagen Endo-free maxi prep kit (Qiagen, Chatsworth, CA). The full-length TcG2 and TcG4 from pCDNA3 were then subcloned into the NTC9385R-MCS nanoplasmid (Nature Technology, Lincoln, NE)^20^. Recombinant nanoplasmids NTC9385R-TcG2 and NTC9385R-TcG4 were sequenced to confirm the orientation and open reading frame, and 1 mg concentrations of each plasmid were purified using the HyperGRO fermentation process^60^. The plasmid map is presented in Fig. 1a.

### Mice, immunization, challenge infection, and mating

C57BL/6 mice (6–8 weeks old) were purchased from Jackson Laboratory (Bay Harbor, ME) and maintained under controlled temperature, humidity, and light cycle conditions according to the requirements of the species, with food and water ad libitum. The schematic view of the study design and sample and data collection is presented in Fig. 1b. Briefly, female mice were randomly distributed in eight groups: (1) vehicle-only/mock infection (C), (2) nano2/4 vaccine (Va), (3) *Trypanosoma cruzi* (*Tc*), (4) nano2/4 followed by *Tc* (VaTc), (5) pregnant (P), (6) nano2/4 before pregnancy (VaP), (7) *Tc* before pregnancy (TcP), (8) nano2/4 followed by *Tc* and pregnancy (VaTcP). We have previously found that DNA vaccine constituted of 25-μg each of pCDNA3-TcG2 and pCDNA3-TcG4 elicited significant protection from *Tc* infection in mice^13,61^. Considering that NTC9385R is more efficacious than pCDNA3 in gene expression, and >50% smaller than pCDNA3 (1753bp vs. 5546 bp), we opted to constitute the nano2/4 vaccine with 12-µg each of NTC9385R-TcG2 and NTC9385R-TcG4 in 50μL 1X PBS and delivered by intramuscular injection in the hind thighs. Prime and booster doses of naon2/4 were given on day 0 and day 21. Challenge with *Tc* trypomastigotes (10,000 parasites per mouse, intraperitoneal) was performed on day 42 ( = 21 days after 2nd vaccine dose). Mice in groups 5–8 were mated on day 49 ( = 7 days post-infection (pi)). C57BL/6 mice have a gestation period of 18 days, and therefore the rationale for choosing 7 days pi was to allow fetal development during the acute parasitemic phase. Harem breeding was used, keeping 4 dams (infected or uninfected) with 1 male (uninfected) mouse, and female mice were monitored daily for the formation of a vaginal plug indicating seminal inoculation (E0 day of gestation). All mice were monitored for physical/clinical well-being, scored as 1 (healthy), 2 (ruffled fur, lethargic), 3 (additional clinical signs of hunched posture, orbital tightening or >15% weight loss), and 4 (score of 3 and reluctance to move when stimulated or >20% weight loss). All mice were euthanized at 61–66 days (=19–24 days pi and E12–17 gestation days). Euthanasia was performed by using inhaled anesthetics (1–4% isoflurane) followed by cervical dislocation to ensure death.

Days paired to fertilization were calculated by subtracting the days paired with males for mating and the approximate day of gestation at harvest for C57BL/6 female mice. Harvesting between E12-E17 gestational days resulted in a variation of placenta weight, fetal weight, and placental efficiency between dams, determined after dissecting the placental and fetal tissues away from uterine tissue. To normalize the data, the coefficient of variation was calculated to determine the dispersion of these parameters between dams for each group. The percent coefficient of variation for the parameters determines that the higher the percent, the greater the dispersion around the mean between the dams. Placental efficiency was calculated as the grams of fetus produced per gram of placenta^62^.

Serum samples from all mice were stored at −20 °C. Heart and skeletal muscle (SkM) tissues from dams and placenta and fetuses from pregnant mice were stored at −80 °C for DNA purification or fixed in 10% buffered formalin for histology. Freshly obtained splenocytes from all dams and placental cells from pregnant dams were used for flow cytometry analysis.

### Serology

Serum antibody response was monitored by enzyme-linked immunosorbent assays (ELISA). Briefly, *Tc* trypomastigotes (1 × 10^10^ trypomastigotes) were pelleted, washed, and suspended in 2 mL 1x phosphate-buffered saline (PBS), and subjected to three freezes (−80 °C, 20 min) – thaw (room temperature, 10 min) cycles. Parasites were centrifuged at 13,500 rpm for 10 min, and the pellet was suspended in a 2 mL RIPA buffer containing protease inhibitors cocktail (Sigma-Aldrich) and sonicated at maximum speed for 5 min in an Ultrasonic bath sonicator (Emerson-Branson). After centrifugation as above, protein concentration in the soluble trypomastigotes lysate (TcL) was measured by Bradford assay (Bio-Rad). The 96-well flat-bottom plates (Corning, RFE 9018) were coated overnight at 4 °C with TcL in PBS (4-μg/100 μL/well). Plates were washed once with PBS-0.05% Tween-20 (PBST, from Invitrogen) using a microplate washer, blocked for 1 h with 1% BSA-PBST, and incubated for 2 h with test sera (1:50 and 1:250 dilutions in 1% BSA-PBST, 100 μL/well). Plates were washed five times as above, and sequential incubations were performed. *Total IgG:* goat anti-mouse IgG (Fc)-biotin (1:10,000 dilution, ab97265, Abcam) for 1 h and streptavidin-horse radish peroxidase (HRP) conjugate (1:10,000 dilution, ab7403, Abcam) for 30 min. *IgG1:* goat anti-mouse IgG1 (1:1000 dilution, ab97236, Abcam) for 1 h, donkey anti-goat IgG-biotin (1:10,000 dilution, ab7124, Abcam) for 1 h, and streptavidin-HRP conjugate (1:10,000 dilution) for 30 min. *IgG2a*: goat anti-mouse IgG2a (1:1000 dilution, ab97241, Abcam), donkey anti-goat IgG-biotin (1:10,000 dilution) for 1 h, and streptavidin-HRP conjugate (1:10,000 dilution) for 30 min. All antibodies were diluted in 1% BSA-PBST. All incubations were performed at 37 °C. The color was developed by incubation with 100 μL/well Sure Blue TMB substrate (T0440, Sigma-Aldrich), the reaction was stopped with 2 N sulfuric acid, and change in color was recorded at 450 nm by using a Multiskan SKY microplate reader (Molecular Devices).

### Flow cytometry

Spleen and placenta samples were macerated, and single-cell suspensions were prepared by standard methods. The placental tissues were digested with 1 mg of collagenase Type II (C2-28, Sigma) in 100μL of Hanks’ Balanced Salt Solution (14025092, ThermoFisher Scientific) for 2 h at 37 °C. Splenocytes were incubated with red blood cell lysis buffer (00-4333-57, eBioscience, San Diego, CA), and washed with cold 1x PBS. Cells (splenocyte and placenta) were counted using a hemocytometer and incubated for 10 min at 4 °C with Fc Block (anti-CD16/CD32; BD Pharmingen, San Diego, CA). Cells (5 × 10^4^ per 50 μL) were washed twice, suspended in 50 µL of flow cytometry staining buffer (00-4222-26, eBioscience), brilliant stain buffer (566385, BD Biosciences, San Jose, CA) and labeled with a cocktail of fluorophore-conjugated cell surface antibodies (concentration determined by titration) for 30 min at 4 °C in dark. Please see Supplementary Table 1 for detailed information on antibodies used in flow cytometry. Cells were washed twice in cold staining buffer, fixed and permeabilized with cytofix/cytoperm solution (BD Biosciences) for 20 min, and washed with perm wash buffer (BD Biosciences). For analyzing intracellular molecules, cells were stained with monoclonal antibodies against cytokines (IFNγ, TNFα, IL10), transcription factor FoxP3, and cytotoxic molecules perforin (PRF1) and granzyme B (GZB) for 30 min, washed, and resuspended in staining buffer.

For studying the recall response, splenocytes were suspended in color-free RPMI media (4 × 10^5^ cells/100μL), and incubated with or without antigenic *Tc* lysate (2.5 μg/100μL) at 37 °C, 5% CO_2_ for 48 h. For the measurement of intracellular cytokines and immune cell activation markers, brefeldin A (10 μg/mL, BD Biosciences) was added to splenocyte cultures for the final 4 h of incubation to block protein secretion. Staining of surface and intracellular molecules was performed as above.

All samples were visualized and analyzed using the 15-color BD LSRII Fortessa flow cytometer. Unstained cells and cells incubated with isotype-matched IgGs (eBioscience), and FMO (fluorescence minus one) were included as controls. Data were analyzed by using FlowJo software (v.10.5.3; TreeStar, San Carlo, CA). Briefly, a concatenated FCS file from all splenic (or placental) samples was generated that was then applied to filter and remove doublet cells, select an average 1×10^5^ of CD3^+^ T cells per sample, and identify T-cell subpopulations using the gating strategy presented in Supplementary Fig. 1. Some of the T-cell subsets were individually analyzed for the expression of intracellular cytokines (IFN-γ, TNFα, or IL10) and cytotoxic (PRF1 or GZB) or regulatory (FOXP3) molecules.

### Cytokines and chemokines

A custom-designed Mouse Luminex Discovery Assay was conducted to measure 21 cytokines and chemokines and other molecules (angiopoietin, CCL2/MCP1, CCL3/MIP1α, CCL5/RANTES, CCL7/MCP3, CCL12/MCP5, CXCL2/MIP2, CXCL10/IP10, CXCL12/SDF1α, ICAM1, IFNγ, IL1β, IL2, IL-4, IL6, IL10, IL17A, IL33, TNFα, uPAR, VEGF) in serum samples of mice from different groups, according to the instructions provided by the manufacturer (R&D Systems, Minneapolis, MN). Briefly, analyte-specific 21 capture antibodies were pre-coated onto magnetic microparticles embedded with fluorophores at set ratios for each unique bead region. Microparticles, standards, and serum samples (100 μL, 1: 10 dilution) were pipetted into wells incubated for 2 h to allow the immobilized antibodies to bind to the analytes in samples. After washing, 50μL of biotinylated detection antibodies cocktail specific to the 21 analytes was added and incubation was performed for 1 h. Following a wash, phycoerythrin-streptavidin conjugate was added, and incubation was performed for 30 min. After the final wash, microparticles were resuspended in a buffer and read using a Luminex 200 analyzer (R&D Systems). In the analyzer, a magnet captures the magnetic microparticles in a monolayer, and two LEDs, one for exciting the dyes inside each bead and a second for phycoerythrin, are used to measure the amount of analyte bound to the bead. Sample from each well was imaged with a CCD camera with a set of filters such that excitation levels and emission levels were differentiated by an in-built photomultiplier tube. A standard curve was prepared using recombinant proteins.

### Parasite burden

Murine maternal and fetal tissues (50 mg each) were homogenized using the Tissue-Tearor (BioSpec Products, Bartlesville, OK), and total DNA was isolated using the DNeasy Blood & Tissue Kit (Qiagen, Chatsworth, CA). Total DNA was examined for quality (OD_260_/OD_280_ ratio: 1.7–2.0) and quantity (1 OD_260_ unit = 50 μg/mL) by using a NanoDrop 2000 spectrophotometer. Total DNA (25 ng) was used as a template with 0.5 μM each of *Tc*18SrDNA-specific (forward: 5′-TTTTGGGCAACAGCAGGTCT-3′, reverse: 5′- CTGCGCCTACGAGACATTCC-3′, amplicon size: 199 bp) and murine Gapdh-specific (forward: 5′-AACTTTGGCATTGTGGAAGG-3′, reverse, 5′-ACACATTGGGGGTAGGAACA-3, amplicon size: 223 bp) oligonucleotides and SYBR Green Supermix (172–5271, Bio-Rad, Hercules, CA) in a final reaction volume of 20 μL. Real-time quantitative PCR was performed for 40 cycles (95 °C for 10 s, 55 °C for 15 s, and 72 °C for 10 s) on an iCycler thermal cycler. Each sample was analyzed in duplicate, and the threshold cycle (*C*_T_) values for *Tc*18SrDNA were normalized to Gapdh reference DNA. The relative parasite burden (*Tc*18SrDNA level) was calculated by following the 2^−ΔΔCT^, where Δ*C*_T_ represents the *C*_T_ (sample) – *C*_T_ (Gapdh) and ΔΔ*C*_T_ represents Δ*C*_T_ (sample) – Δ*C*_T_ (no infection control). Tissues were considered positive for *Tc*18SrDNA with 95% confidence that had a *C*_T_ value less than [mean *C*_T_ for no infection control – (1.95 for *z*-score) x (standard deviation for no infection control *C*_T_)].

### Histology

Maternal (heart, SkM) and fetal (placenta) tissue sections were fixed in 10% buffered formalin, dehydrated in graded ethyl alcohol, cleared in xylene, and embedded in paraffin. Paraffin-embedded 5-micron tissue sections were stained with hematoxylin and eosin (H&E) or Masson’s trichrome. Slides (at least two per tissue, nine microscopic fields per slide) were imaged at 40x (cardiac and SkM) and 20x (placenta) magnification with an Olympus BX-15 microscope (Center Valley, PA) equipped with a digital camera and Simple PCI software (v.6.0, Compix, Sewickley, PA). Tissue sections stained with H&E were scored for inflammation (i.e., % nuclei) and necrosis (i.e., % tears in tissue). ImageJ version 1.53 (last accessed on Dec 15, 2022, https://imagej.nih.gov/ij/index.html) was used to quantify pixels with nuclei coloration and tissue tears. Briefly, total tissue pixels and nuclei were selected for using color thresholding and percent nuclei quantified [(nuclei pixels ÷ total tissue pixels) x 100]. For the quantification of percent tears [(tears in tissue pixels ÷ total tissue pixels) x 100], total tissue pixels and tears in tissue were selected for using thresholding. Cardiac, SkM, and placental tissue sections stained with Masson’s trichrome were scored for percent fibrotic tissue using ImageJ to quantify fibrotic (blue) pixels [(blue pixels ÷ total tissue pixels) x 100].

### Statistical analysis

Data were assembled in Excel and analyzed by using GraphPad Prism v.9.4.0 (San Diego, CA). Shapiro-Wilk normality test was performed to check the normal distribution of data. Significance (*p*-values) between the groups (control vs. vaccinated, infected, or vaccinated/infected; infected vs. vaccinated/infected; and vaccinated vs. vaccinated/infected; with or without pregnancy) was calculated by Students’ unpaired *t*-test (or paired *t*-test for recall response) with or without Welch’s correction or Mann–Whitney *U*-test (or Wilcoxon test for recall response). Detailed information including mean values, standard error mean (SEM), *p*-values (*p < 0.05, ** *p* < 0.01, ***p < 0.001, ****p < 0.0001), and data collected from all groups of mice are shown in Supplementary Tables 2–8. In graphs, mean values, SEM, and significance are plotted.

### Supplementary information

Supplemental material

## Data Availability

All data are provided within the manuscript.
