# Supplementary material for: Subunit nanovaccine elicited T cell functional activation controls Trypanosoma cruzi mediated maternal and placental tissue damage and improves pregnancy outcomes in mice
Source: NPJ Vaccines. 2023 Dec 16;8:188. doi: 10.1038/s41541-023-00782-z (PMC10725459; doi:10.1038/s41541-023-00782-z)
Supplement: Supplementary file 1 — Supplemental material [file 41541_2023_782_MOESM1_ESM.pdf]

SUPPLEMENTAL FILE

Subunit nanovaccine elicited T cell functional activation controls *Trypanosoma cruzi* mediated maternal and placental tissue damage and improves pregnancy outcomes in mice.

Lizette Elaine Rios [1, 2], Nandadeva Lokugamage [1], Subhadip Choudhuri [1], Imran Hussain Chowdhury [1], Nisha Jain Garg [1, 3, 4]

[1] Department of Microbiology and Immunology, University of Texas Medical Branch (UTMB), Galveston, TX

[2] Department of Biochemistry and Molecular Biology, UTMB, Galveston, TX

[3] Institute for Human Infections and Immunity (IHII), UTMB, Galveston, TX

[4] Sealy Institute for Vaccine Sciences (SIVS), UTMB, Galveston, TX

Running title: Vaccine efficacy against *Tc* in pregnancy.

Corresponding author: Dr. Nisha J Garg, Department of Microbiology & Immunology, University of Texas Medical Branch, 301 University Boulevard, Galveston, TX, USA, 77555-1070. E: nigarg@utmb.edu

Supplementary Table 1. Flow cytometry reagents.

**Antigen**	Fluorophore	Anti-mouse antibody, catalog#, dilutions	Source
**A. Antibodies used for splenic T cells analysis**			
CD3	APC	Hamster IgG1, κ, 553066, 1: 100	BD Bioscience
CD4	BV510	Rat DA/HA IgG2a, κ, 563106, 1: 100	BD Horizon
CD8a	BUV395	Rat LOU/C, LOU/M IgG2a, κ, 563786, 1: 100	BD Horizon
CD25	BV421	Rat LEW, IgM, κ, 564571, 1: 100	BD Horizon
CD62L	BV650	Rat F344, CDF IgG2a, κ, 564108, 1: 100	BD Horizon
CD44	BV786	Rat IgG2b, κ, 563736, 1: 100	BD Horizon
Granzyme B (GZB)	PE	Rat / IgG2a, κ, 12-8898-82, 1: 100	Invitrogen
Perforin (PRF1)	FITC	Rat / IgG2a, κ, 11-9392-82, 1: 100	Invitrogen
IFNγ	BV711	Rat IgG1, κ, 564336, 1: 100	BD Horizon
**B. Antibodies used for placental T cells analysis**			
CD3	FITC	Hamster IgG1 κ, 553062, 1: 100	BD Pharmingen
CD4	BV510	Rat DA/HA IgG2a, κ, 563106, 1: 100	BD Horizon
CD8a	BUV395	Rat LOU/C, LOU/M IgG2a, κ, 563786, 1: 100	BD Horizon
CD25	BV421	Rat LEW, IgM, κ, 564571, 1: 100	BD Horizon
CD45	BV786	Rat LOU/C, LOU/M IgG2b, κ, 564225, 1: 100	BD Horizon
CD62L	BV650	Rat F344, CDF IgG2a, κ, 564108, 1: 100	BD Horizon
CD44	APC	Rat / IgG2b, κ, 17-0441-82, 1: 100	Thermo Fisher
TNFα	PerCP-Cy 5.5	Rat IgG1, 560659, 1: 100	BD Pharmingen
FOXP3	PE-CF594	Rat IgG2b, κ, 562466, 1: 100	BD Horizon
IL10	PE-Cy7	Rat IgG2b, κ, 505026, 1: 100	BioLegend
IFNγ	APC efluor 780	Rat IgG1, κ, 47-7311-82, 1: 100	Invitrogen
**C. Phenotypic profile of T cell subsets**			
Double negative (DN)		CD3+CD4-CD8-	
Double positive (DP)		CD3+CD4+CD8+	
CD4+		CD3+CD4+CD8-	
CD4+ T naive (Tnv)		CD3+CD4+CD8-CD25-CD62L++CD44-	
CD4+ T effector / effector memory (Tem)		CD3+CD4+CD8-CD25-CD62L-CD44++	
CD4+ T central memory (Tcm)		CD3+CD4+CD8-CD25-CD62L+CD44+	
CD4+ T regulatory (Treg)		CD3+CD4+CD8- CD62L+CD44+ CD25+ or<br>CD3+CD4+CD8- CD62L+CD44+ CD25+FOXP3+	
CD8+		CD3+CD4-CD8+	
CD8+ Tnv		CD3+CD4-CD8+CD25-CD62L++CD44-	
CD8+ Tem		CD3+CD4-CD8+CD25-CD62L-CD44++	
CD8+ Tcm		CD3+CD4-CD8+CD25-CD62L+CD44+	

Antibodies used for flow cytometry analysis of splenic **(A)** and placental **(B)** T cells are listed. Clustering of the T cell subsets based on the surface expression of several markers is listed in **C.** The Tnv, Tem, Tcm, and Treg subsets were analyzed for the expression levels of intracellular cytokines (TNFα, IFNγ, or IL10), cytotoxic molecules (PRF1, GZB) and FOXP3.

Supplementary Table 2. Vaccine induced phenotypic and functional profile of splenic T cell subsets in pregnancy.

Subpopulations	Values	Control n=8	Vaccine (Va) n=6	Pregnant (P) n=6	VaP n=6	Stats: P values / Test applied			
						C vs P	C vs Va	P vs VaP	Va vs VaP
**Ex vivo phenotypic profile of splenic T cells (Percent frequency of parent populations)**									
CD4-CD8-	Mean	14.700	13.300	18.960	12.260	0.0183	0.7546	0.2208	0.9999
	SEM	8.690	4.780	4.620	5.720	MW	MW	MW	MW
CD4+CD8+	Mean	0.270	0.000	0.400	0.390	0.2601	0.7266	0.9541	0.4545
	SEM	0.050	0.000	0.110	0.060	U	MW	U	MW
CD4+	Mean	51.600	63.000	48.400	59.600	0.1079	0.3450	0.0787	0.6540
	SEM	5.390	5.000	2.840	4.960	MW	MW	U	U
CD4+ Tnv	Mean	70.700	63.000	65.010	55.000	0.1079	0.2284	0.4134	0.5401
	SEM	5.960	5.000	3.100	10.000	MW	MW	U	U
CD4+CD25+	Mean	13.400	13.970	11.070	12.130	0.0186	0.7682	0.5628	0.3636
	SEM	0.830	1.750	0.550	0.840	MW	U	MW	U
CD4+ Tem	Mean	14.900	16.000	16.000	19.500	0.7861	0.6844	0.3762	0.4240
	SEM	2.950	1.000	1.460	3.560	U	W	U	W
CD4+ Tcm	Mean	4.050	7.000	5.010	5.920	0.0813	0.0127	0.2456	0.3297
	SEM	0.730	1.000	0.360	0.650	MW	MW	U	U
CD8+	Mean	33.400	23.500	32.200	27.800	0.4908	0.0293	0.0411	0.5887
	SEM	3.730	2.330	2.550	2.820	MW	MW	MW	MW
CD8+ Tnv	Mean	75.200	45.000	76.800	65.400	0.4908	0.0200	0.9999	0.3095
	SEM	6.420	3.000	3.400	10.800	MW	MW	MW	MW
CD8+CD25+	Mean	0.260	0.000	0.250	0.090	0.8483	0.2535	0.0051	0.0600
	SEM	0.050	0.000	0.040	0.010	U	U	W	U
CD8+ Tem	Mean	3.020	7.000	2.330	3.990	0.8518	0.0200	0.2214	0.1505
	SEM	1.100	2.000	0.400	1.150	MW	MW	W	U
CD8+ Tcm	Mean	11.600	32.000	15.200	8.420	0.0798	0.0058	0.0051	0.0029
	SEM	1.190	5.000	1.480	1.190	U	W	U	W
**Ex vivo functional activation profile of splenic T cells (Percent frequency of parent populations)**									
CD4+ Tnv IFNγ	Mean	0.3215	0.355	0.265	1.149	0.4840	0.5821	0.0087	0.0087
	SEM	0.047	0.030	0.064	0.483	U	U	MW	MW
CD4+ Tnv PRF1	Mean	0.275	0.154	0.133	1.138	0.0139	0.0388	0.0022	0.0022
	SEM	0.040	0.028	0.020	0.510	U	U	MW	MW
CD4+ Tnv GZB	Mean	0.570	0.383	0.372	0.658	0.2819	0.1986	0.2053	0.1971
	SEM	0.131	0.019	0.103	0.184	U	W	U	W
CD4+ Tem IFNγ	Mean	0.408	0.268	0.420	0.391	0.9014	0.1343	0.6593	0.0251
	SEM	0.071	0.032	0.054	0.034	U	U	U	U
CD4+ Tem PRF1	Mean	0.209	0.094	0.186	0.281	0.3606	0.0075	0.1126	0.0006
	SEM	0.028	0.019	0.028	0.033	MW	U	MW	U
CD4+ Tem GZB	Mean	0.656	0.411	0.500	0.658	0.3997	0.1604	0.3904	0.1429
	SEM	0.130	0.073	0.110	0.137	U	U	U	U
CD4+ Tcm IFNγ	Mean	0.271	0.393	0.462	2.319	0.1419	0.2284	0.0043	0.0022
	SEM	0.958	0.049	0.101	1.056	MW	MW	MW	MW
CD4+ Tcm PRF1	Mean	0.242	0.380	0.186	2.425	0.2536	0.1252	0.0022	0.0022
	SEM	0.036	0.085	0.024	1.044	U	U	MW	MW
CD4+ Tcm GZB	Mean	0.743	0.365	0.568	0.791	0.3279	0.0330	0.1069	0.0015
	SEM	0.128	0.058	0.099	0.791	U	U	U	U
CD8+ Tnv IFNγ	Mean	0.310	0.400	0.320	0.310	0.8937	0.1710	0.8240	0.0729
	SEM	0.047	0.026	0.041	0.036	U	U	U	U
CD8+ Tnv PRF1	Mean	0.279	0.130	0.300	0.150	0.1812	0.0320	0.0043	0.8939
	SEM	0.078	0.023	0.033	0.022	MW	MW	MW	MW
CD8+ Tnv GZB	Mean	0.667	0.230	0.480	0.360	0.5728	0.0224	0.0931	0.6991
	SEM	0.149	0.041	0.084	0.120	MW	W	MW	MW
CD8+ Tem IFNγ	Mean	0.488	0.310	0.410	0.550	0.1229	0.0204	0.9805	0.0584
	SEM	0.088	0.048	0.055	0.044	W	U	MW	MW
CD8+ Tem PRF1	Mean	0.311	0.140	0.220	0.340	0.4798	0.1307	0.0671	0.0041
	SEM	0.051	0.029	0.017	0.134	U	U	U	U
CD8+ Tem GZB	Mean	0.955	0.650	1.020	1.380	0.8682	0.2927	0.5016	0.1782
	SEM	0.257	0.106	0.238	0.465	U	W	U	W
CD8+ Tcm IFNγ	Mean	0.332	0.380	0.430	0.400	0.3426	0.5242	0.6951	0.8639
	SEM	0.077	0.031	0.064	0.059	U	W	U	U
CD8+ Tcm PRF1	Mean	0.254	0.110	0.270	0.140	0.6946	0.0054	0.0411	9999
	SEM	0.027	0.031	0.034	0.043	U	U	MW	MW

CD8+ Tcm GZB	Mean	0.839	0.390	0.620	0.380	0.3268	0.0137	0.1201	0.9372
	SEM	0.159	0.074	0.129	0.054	U	MW	U	MW

Female C57BL/6 mice were immunized with nano2/4, mated with males at 28 days after 2nd vaccine dose, and pregnant females were euthanized at E12-17 gestation days. Included as controls were age-matched, non-vaccinated and vaccinated (but not mated) mice. The splenocytes were labeled with fluorophore-conjugated antibodies. CD3^{+}T cells, based on the expression levels of CD4, CD8, CD62L, and CD44, were acquired analyzed by flow cytometry and data were analyzed by FlowJo software. Shown are percent frequencies of CD4^{-}CD8^{-}, CD4+CD8+ T lymphocytes, and CD4^{+} and CD8^{+} T cell subsets that exhibited naïve (Tnv), effector/effector memory (Tem), and central memory (Tcm) phenotypes and produced IFNγ, Perforin (PRF1) or Granzyme B (GZB) molecules. Mean ± SEM values derived from duplicate observations per sample (n=6-8 mice per group) are shown. Significance was calculated by Students' unpaired t test (U) with or without Welch's correction (W) or Mann-Whitney U test (MW) and p values at <0.05 are underlined. These data are presented in **Figure 2**.

Supplementary Table 3. Recall response of vaccine induced splenic T cells in pregnancy.

Subpopulations	Values	Control (C)		Vaccine (Va)		Pregnant (P)		VaP		Stats: P values / Test applied for +TcL			
		-TcL (n=8)	+TcL(n=6)	-TcL(n=6)	+TcL(n=6)	-TcL(n=6)	+TcL(n=6)	-TcL(n=6)	+TcL(n=6)	C vs P	C vs Va	P vs VaP	Va vs VaP
**Phenotypic profile of splenic T cells after in vitro stimulation with *Tc* lysate (Percentages of parent populations)**													
CD4-CD8-	Mean	10.21	6.863	14.1	22.840	14.48	19.000	10.4	10.34	0.0083	0.1153	0.0279	0.1993
	SEM	2.755	0.850	3.35	8.380	1.495	3.000	2.55	1.640	W	W	U	W
CD4+CD8+	Mean	0.954	0.890	1.688	0.838	0.743	0.146	1.451	2.998	0.0191	0.6991	0.0844	0.1797
	SEM	0.173	0.142	0.292	0.186	0.108	0.046	0.223	1.202	W	MW	W	MW
CD4+	Mean	48.81	53.370	44.750	39.400	48.430	46.820	49.580	51.460	0.0046	0.0931	0.0389	0.5887
	SEM	1.437	0.886	2.746	7.666	1.158	1.572	2.553	1.160	U	MW	U	MW
CD4+Tnv	Mean	23.18	30.350	5.31	9.540	31.28	38.760	2.37	8.330	0.1751	0.0038	<0.0001	0.7055
	SEM	2.297	5.111	0.976	2.201	4.318	2.661	0.269	2.178	U	U	U	U
CD4+CD25+	Mean	7.04	8.170	7.01	6.510	8.23	12.230	5.23	9.570	0.0411	0.2835	0.2403	0.0937
	SEM	0.948	1.176	0.616	0.868	1.702	1.458	0.604	1.406	MW	U	MW	U
CD4+Tem	Mean	15.96	16.260	18.03	22.680	17.52	19.99	19.36	25.900	0.2222	0.0278	0.1446	0.3866
	SEM	1.107	2.204	1.266	1.178	1.583	1.836	1.831	3.251	U	U	U	W
CD4+Tcm	Mean	1.75	2.150	2.85	2.490	2.24	2.430	2.18	3.340	0.595	0.6884	0.4088	0.4964
	SEM	0.158	0.426	0.656	0.691	0.278	0.264	0.600	0.987	U	U	W	U
CD8+	Mean	40.020	38.880	39.520	36.920	36.360	33.630	38.540	35.210	0.0234	0.3187	0.5475	0.5028
	SEM	1.517	1.191	1.119	1.437	0.871	1.558	1.217	1.954	U	U	U	U
CD8+Tnv	Mean	13.070	22.620	1.835	2.658	24.770	30.630	1.138	1.821	0.3213	0.0219	0.0016	0.326
	SEM	1.982	6.109	0.163	0.747	5.086	4.694	0.237	0.233	U	W	W	W
CD8+CD25+	Mean	6.209	2.632	4.003	2.017	15.380	7.874	2.458	1.230	0.0411	0.5887	0.0077	0.0947
	SEM	3.233	1.226	0.885	3.777	2.718	1.550	0.502	0.134	MW	MW	W	W
CD8+Tem	Mean	18.670	16.080	14.200	17.070	19.230	16.380	18.110	21.880	0.8918	0.7178	0.0649	0.0584
	SEM	1.426	1.575	0.897	2.196	1.589	1.609	2.826	1.702	U	U	MW	MW
CD8+Tcm	Mean	1.163	1.538	2.545	2.002	2.198	2.155	1.743	2.458	0.1881	0.5031	0.6672	0.603
	SEM	0.175	0.381	0.775	0.548	0.354	0.214	0.503	0.649	U	U	W	U
**Functional activation profile of splenic T cells after in vitro stimulation with *Tc* lysate (Percentages of parent populations)**													
CD4+Tnv IFNγ	Mean	0.386	0.546	1.753	1.692	0.606	0.604	4.399	5.024	0.5764	0.0005	0.0007	0.0145
	SEM	0.043	0.057	0.293	0.166	0.056	0.084	0.782	0.920	U	W	U	W
CD4+Tnv PRF1	Mean	0.457	0.651	2.260	1.790	0.538	0.639	5.679	8.429	0.8673	0.0079	0.0301	0.0507
	SEM	0.025	0.045	0.377	0.270	0.039	0.051	1.326	2.507	U	W	W	W
CD4+Tnv GZB	Mean	0.586	0.643	0.457	1.492	0.460	0.608	2.225	4.877	0.6474	0.0003	0.0176	0.0395
	SEM	0.048	0.059	0.043	0.143	0.022	0.045	0.218	1.225	U	U	W	W
CD4+Tem IFNγ	Mean	0.541	0.748	0.578	0.445	0.639	0.677	0.716	1.546	0.6069	0.0458	<0.0001	<0.0001
	SEM	0.061	0.120	0.088	0.580	0.058	0.059	0.037	0.077	U	U	U	U
CD4+Tem PRF1	Mean	0.481	0.799	1.558	0.478	0.528	0.723	0.510	7.103	0.6425	0.031	0.0824	0.0742
	SEM	0.037	0.108	0.251	0.069	0.083	0.116	0.040	2.943	U	U	W	W
CD4+Tem GZB	Mean	0.875	1.003	0.618	0.515	0.568	0.719	0.723	0.610	0.0159	0.0041	0.1048	0.3961
	SEM	0.035	0.089	0.082	0.097	0.029	0.042	0.053	0.045	U	U	U	U
CD4+Tcm IFNγ	Mean	0.539	0.917	0.668	2.060	0.517	2.354	1.001	1.526	0.0007	0.0008	0.0536	0.144
	SEM	0.139	0.184	0.088	0.160	0.063	0.236	0.106	0.297	U	U	U	U
CD4+Tcm PRF1	Mean	0.764	1.400	0.553	1.182	0.813	3.066	1.695	3.011	0.0115	0.5777	0.1009	0.0626

	SEM	0.102	0.333	0.088	0.181	0.153	0.424	0.147	0.535	U	U	U	W
CD4+Tcm GZB	Mean	0.977	1.449	0.870	1.123	0.428	2.438	0.954	7.213	0.0931	0.0649	0.0854	0.2403
	SEM	0.103	0.059	0.139	0.141	0.037	0.342	0.097	2.230	MW	MW	W	MW
CD8+Tnv IFNγ	Mean	0.295	0.567	0.63	0.55	0.44	0.480	1.04	0.620	0.4848	0.9035	0.3095	0.6556
	SEM	0.034	0.078	0.057	0.092	0.045	0.062	0.237	0.119	MW	U	MW	U
CD8+Tnv PRF1	Mean	0.388	0.613	0.50	0.46	0.37	0.490	0.74	0.620	0.1493	0.1701	0.1046	0.1413
	SEM	0.038	0.052	0.078	0.089	0.032	0.058	0.161	0.038	U	U	U	U
CD8+Tnv GZB	Mean	0.535	0.586	0.53	0.58	0.48	0.420	0.87	0.710	0.026	0.7857	0.1032	0.4242
	SEM	0.069	0.047	0.037	0.076	0.047	0.037	0.133	0.145	MW	MW	W	U
CD8+Tem IFNγ	Mean	0.528	0.706	0.47	0.45	0.48	0.670	0.70	0.750	0.1585	0.0897	0.1994	0.0104
	SEM	0.060	0.123	0.071	0.021	0.053	0.037	0.034	0.031	W	W	U	U
CD8+Tem PRF1	Mean	0.335	0.459	0.35	0.35	0.26	0.330	0.52	0.500	0.2228	0.2989	0.0363	0.0623
	SEM	0.034	0.09	0.013	0.047	0.038	0.045	0.026	0.035	U	U	U	U
CD8+Tem GZB	Mean	0.731	0.802	0.60	0.62	0.48	0.610	0.72	0.540	0.2228	0.2298	0.408	0.3853
	SEM	0.083	0.126	0.070	0.07	0.035	0.071	0.050	0.043	U	U	U	U
CD8+Tcm IFNγ	Mean	0.324	0.648	0.25	0.4	0.58	0.490	0.82	0.600	0.1212	0.0714	0.1212	0.132
	SEM	0.034	0.076	0.026	0.084	0.069	0.105	0.183	0.072	MW	MW	MW	MW
CD8+Tcm PRF1	Mean	0.353	0.483	0.36	0.416	0.58	1.040	0.98	0.640	0.0045	0.7527	0.0404	0.1505
	SEM	0.042	0.067	0.018	0.056	0.063	0.136	0.158	0.102	U	U	U	U
CD8+Tcm GZB	Mean	0.601	0.749	0.58	0.56	0.50	0.700	1.05	0.560	0.7169	0.1628	0.1601	0.9595
	SEM	0.056	0.104	0.021	0.067	0.027	0.066	0.309	0.069	U	U	U	U

Female mice (C57BL/6) were given empty plasmid or nano2/4 vaccine, then mated with males at 28 days post 2nd vaccine dose and euthanized at E12-17 gestation days. The age-matched, non-vaccinated and vaccinated (but not mated) mice were included as controls. Splenocytes were *in vitro* stimulated for 48 h with or without antigenic *Tc* lysate (TcL). Cells were then labeled with fluorophore-conjugated antibodies and analyzed by flow cytometry equipped with FlowJo software. Percent frequencies of CD4$^-$CD8$^-$, CD4+CD8+ T lymphocytes, and CD4$^+$ and CD8$^+$ T cell subsets that exhibited naïve (Tnv), effector/effector memory (Tem), and central memory (Tcm) phenotypes are shown. The frequencies of IFNγ, perforin (PRF1) and granzyme (GZB) expressing T cell subsets are also shown. Mean ± SEM values were derived from duplicate observations per sample (n=6-8 mice per group). Significance was calculated by Students' unpaired t test (U) with or without Welch's correction (W) or Mann-Whitney U test (MW) and p values at <0.05 are underlined. These data are presented in **Supplementary Figure 2.**

Supplementary Table 4. Expansion of vaccine induced splenic T cell response after challenge *T. cruzi* infection in pregnant mice.

Parameter	Values	Control n=8	*T. cruzi* n=12	Vaccine (Va) Tc n=13	Pregnant (P) n=6	TcP n=10	VaTcP n=6	Statistical analysis: P values / Test applied					
								C vs Tc	Tc vs VaTc	P vs TcP	TcP vs VaTcP	Tc vs TcP	VaTc vs VaTcP
**Ex vivo phenotypic profile of splenic T cells (Percentages of parent population)**													
CD4-CD8-	Mean	14.710	25.440	8.990	18.960	26.570	7.220	0.0691	0.0132	0.5441	0.0048	0.8848	0.4121
	SEM	8.690	5.530	1.260	4.620	5.220	1.430	MW	W	MW	W	U	MW
CD4+CD8+	Mean	0.270	0.530	0.930	0.400	1.000	0.570	0.0228	0.1004	0.0934	0.7925	0.3716	0.1582
	SEM	0.050	0.080	0.140	0.110	0.000	0.050	U	MW	MW	MW	MW	MW
CD4	Mean	51.600	37.700	45.900	48.400	41.000	65.300	0.0041	0.1519	0.1769	0.0004	0.3442	0.0032
	SEM	5.390	1.970	3.930	2.840	3.000	3.000	MW	MW	U	U	U	MW
CD4+Tnv	Mean	70.700	33.960	38.100	65.010	46.400	44.600	0.0010	0.3684	0.0075	0.7739	0.0237	0.1368
	SEM	5.960	3.840	2.120	3.100	3.810	4.150	MW	MW	MW	U	MW	U
CD4+Treg	Mean	13.440	6.180	8.600	11.070	6.320	5.840	<0.0001	0.0047	0.0002	0.9999	0.6744	0.7012
	SEM	0.830	0.570	0.620	0.550	0.190	2.150	MW	MW	MW	MW	MW	MW
CD4+Tem	Mean	14.900	47.200	43.300	16.000	29.000	36.300	<0.0001	0.1722	0.0008	0.1037	0.0003	0.0432
	SEM	2.950	3.030	1.490	1.460	2.000	3.530	MW	MW	U	U	MW	U
CD4+Tcm	Mean	4.050	7.170	6.950	5.010	6.000	6.800	0.0055	0.3760	0.3329	0.1404	0.0425	0.3676
	SEM	0.730	0.520	0.610	0.360	0.000	0.410	MW	MW	U	U	MW	MW
CD8+	Mean	33.400	36.400	44.200	32.200	31.400	26.900	0.9101	0.1519	0.8176	0.2246	0.3495	0.0220
	SEM	3.730	4.660	5.070	2.550	2.170	2.890	MW	MW	U	U	W	MW
CD8+Tnv	Mean	75.200	24.300	30.800	76.800	39.000	21.400	<0.0001	0.6395	<0.0001	0.0075	0.0160	0.3229
	SEM	6.420	3.180	5.890	3.400	5.000	2.950	MW	MW	U	MW	MW	MW
CD8+Treg	Mean	0.260	0.530	1.180	0.250	1.000	0.270	0.0417	0.0038	0.0024	0.0104	0.6138	0.0001
	SEM	0.050	0.110	0.160	0.040	0.000	0.080	W	U	U	U	U	W
CD8+Tem	Mean	3.020	48.100	39.600	2.330	30.000	54.600	<0.0001	0.5743	<0.0001	0.0033	0.0082	0.5214
	SEM	1.100	4.440	7.250	0.400	4.000	6.070	MW	MW	W	U	U	MW
CD8+Tcm	Mean	11.600	15.600	19.100	15.200	16.000	11.300	0.2083	0.9362	0.6917	0.0271	0.3463	0.2100
	SEM	1.190	2.090	2.800	1.480	1.000	1.450	MW	MW	U	U	MW	MW
**Ex vivo functional activation profile of splenic T cells (Percentages of parent population)**													
CD4+Tnv IFNγ	Mean	0.322	0.393	0.469	0.265	0.381	0.341	0.3755	0.2967	0.1114	0.4102	0.9356	0.1262
	SEM	0.047	0.056	0.044	0.064	0.060	0.068	U	U	MW	MW	MW	U
CD4+Tnv PRF1	Mean	0.276	0.389	0.305	0.133	0.353	0.401	0.0314	0.0043	0.0016	0.4278	0.2091	0.1857
	SEM	0.040	0.030	0.011	0.020	0.050	0.065	MW	MW	W	MW	MW	MW
CD4+Tnv GZB	Mean	0.570	0.626	0.563	0.372	0.568	0.474	0.7220	0.8938	0.1207	0.1897	0.5539	0.3783
	SEM	0.131	0.091	0.071	0.103	0.032	0.070	U	MW	W	U	W	MW
CD4+Tem IFNγ	Mean	0.408	0.616	0.532	0.420	0.249	0.513	0.0453	0.3403	0.0074	<0.0001	<0.0001	0.8321
	SEM	0.071	0.062	0.057	0.054	0.028	0.040	MW	MW	U	U	MW	U
	Mean	0.209	0.303	0.265	0.186	0.201	0.378	0.0195	0.2472	0.4734	0.0002	0.0049	0.0071

CD4+Tem PRF1	SEM	0.028	0.023	0.021	0.028	0.021	0.028	U	U	MW	U	U	U
CD4+Tem GZB	Mean	0.656	1.218	0.089	0.500	0.542	1.038	0.1749	0.8406	0.7266	0.0046	0.0116	0.4405
	SEM	0.130	0.285	0.118	0.110	0.027	0.106	MW	MW	W	W	MW	U
CD4+Tcm IFNγ	Mean	0.271	0.512	0.494	0.462	0.783	0.743	0.0658	0.8651	0.1882	0.3676	0.2754	0.0489
	SEM	0.058	0.091	0.062	0.101	0.224	0.110	MW	U	MW	U	MW	U
CD4+Tcm PRF1	Mean	0.242	0.541	0.334	0.186	0.916	0.467	0.0007	0.0012	0.0002	0.0155	0.0161	0.0608
	SEM	0.036	0.054	0.020	0.024	0.219	0.062	U	U	MW	MW	MW	MW
CD4+Tcm GZB	Mean	0.743	1.158	1.527	0.568	1.118	0.743	0.0102	0.0415	0.0225	0.0934	0.1593	0.0035
	SEM	0.128	0.082	0.147	0.099	0.219	0.110	U	W	MW	MW	MW	U
CD8+Tnv IFNγ	Mean	0.310	0.400	0.420	0.320	0.310	0.310	0.2334	0.904	0.9318	0.9269	0.152	0.1342
	SEM	0.047	0.058	0.035	0.040	0.034	0.069	U	U	U	U	W	U
CD8+Tnv PRF1	Mean	0.279	0.250	0.360	0.300	0.200	0.310	0.7921	0.0610	0.0194	0.2864	0.2864	0.5932
	SEM	0.078	0.051	0.028	0.033	0.021	0.089	MW	U	U	W	W	W
CD8+Tnv GZB	Mean	0.667	0.610	0.670	0.480	0.430	0.470	0.8658	0.5382	0.9371	0.4135	0.9999	0.0630
	SEM	0.149	0.112	0.092	0.089	0.032	0.037	MW	MW	MW	U	MW	W
CD8+Tem IFNγ	Mean	0.488	0.810	0.740	0.410	0.560	0.720	0.2785	0.6584	0.5797	0.0005	0.0215	0.0004
	SEM	0.088	0.082	0.034	0.055	0.040	0.049	MW	MW	MW	MW	MW	U
CD8+Tem PRF1	Mean	0.311	0.410	0.340	0.220	0.260	0.540	0.0169	0.4187	0.0320	0.0321	0.0143	0.6834
	SEM	0.051	0.058	0.025	0.017	0.026	0.540	U	W	U	U	W	U
CD8+Tem GZB	Mean	0.955	4.230	1.010	1.020	2.900	6.600	0.0473	0.0305	0.0010	0.2198	0.3463	0.05
	SEM	0.257	1.296	0.116	0.238	0.231	2.225	MW	W	MW	MW	MW	W
CD8+Tcm IFNγ	Mean	0.332	0.400	0.440	0.430	0.320	0.530	0.3559	0.6944	0.0778	0.0051	0.1137	0.3194
	SEM	0.077	0.046	0.053	0.064	0.025	0.072	U	U	U	U	U	U
CD8+Tcm PRF1	Mean	0.255	0.220	0.310	0.270	0.230	0.490	0.2834	0.0233	0.3643	0.0355	0.6590	0.0347
	SEM	0.027	0.022	0.033	0.034	0.027	0.092	U	U	U	W	U	U
CD8+Tcm GZB	Mean	0.839	1.350	0.950	0.620	0.830	1.460	0.1359	0.1933	0.1691	0.4439	0.0980	0.6544
	SEM	0.159	0.283	0.073	0.129	0.046	0.523	W	W	W	MW	W	MW

Mice (C57BL/6, female) were immunized with empty plasmid or nano2/4, challenged with *T. cruzi* at 21 days post second vaccine dose, mated with males at seven days post-infection, and euthanized at E12-17 gestation days. Control mice included non-vaccinated, infected, and vaccinated/infected (but not mated). Splenocytes were labeled with fluorophore-conjugated antibodies. $CD3^+$T cells based on the expression levels of CD4, CD8, CD62L, and CD44 antigens were acquired by flow cytometry and analyzed by FlowJo software. Percent frequencies of $CD4^-CD8^-$, CD4+CD8+ T lymphocytes, and $CD4^+$ and $CD8^+$ T cell subsets that exhibited naïve (Tnv), effector / effector memory (Tem), and central memory (Tcm) phenotypes and produced IFNγ, PRF1 and GZB molecules are shown. Mean ± SEM values were derived from duplicate observations per sample (n=6-13 per group). Significance was calculated by Students' unpaired t test (U) with or without Welch's correction (W) or Mann-Whitney U test (MW) and p values at <0.05 are underlined. Data are presented in **Figure 3.**

Supplementary Table 5. Recall response of vaccine induced splenic T cells in *T. cruzi* infected pregnant mice.

Subsets	Values	Control		*T. cruzi* (Tc)		Vaccine (Va) Tc		Pregnant (P)		TcP		VaTcP		Stats: P values / Test applied for +TcL					
		-TcL (n=8)	+TcL (n=6)	-TcL (n=12)	+TcL (n=12)	-TcL (n=12)	+TcL (n=10)	-TcL (n=6)	+TcL (n=6)	-TcL (n=10)	+TcL (n=10)	-TcL (n=6)	+TcL (n=6)	C vs Tc	Tc vs VaTc	P vs TcP	TcP vs VaTcP	Tc vs TcP	VaTc vs VaTcP
**Phenotypic profile of splenic T cells after in vitro stimulation with *Tc* lysate (Percentages of parent population)**																			
CD4-CD8-	Mean	10.21	6.863	12.89	13.860	14.5	10.330	14.48	19.000	16.08	15.660	9.89	12.770	0.0061	0.1593	0.3676	0.0934	0.6744	0.2198
	SEM	2.755	0.850	2.317	1.490	1.939	1.080	1.495	3.000	3.91	1.970	2.176	5.950	U	MW	MW	MW	MW	MW
CD4+CD8+	Mean	0.954	0.890	1.139	0.979	1.018	1.507	0.743	0.146	5.7750	3.999	0.843	0.608	0.6221	0.1089	0.0005	0.0047	0.002	0.0516
	SEM	0.173	0.142	0.216	0.103	0.091	0.285	0.108	0.046	1.322	0.887	0.300	0.262	U	W	MW	MW	MW	MW
CD4+	Mean	48.81	53.370	44.960	43.760	47.120	53.270	48.430	46.820	44.060	44.750	51.690	51.540	0.0107	0.0481	0.445	0.0934	0.7951	0.9794
	SEM	1.437	0.886	3.293	3.089	4.389	3.277	1.158	1.572	2.506	1.792	1.278	5.919	W	U	U	MW	U	MW
CD4+Tnv	Mean	23.18	30.350	26.98	29.300	19.64	25.720	31.28	38.760	31.85	38.450	3.78	7.280	0.8926	0.574	0.5622	0.0002	0.1272	0.0035
	SEM	2.297	5.110	2.627	4.728	2.627	3.852	4.318	2.661	5.204	3.999	2.380	1.984	U	U	MW	MW	MW	U
CD4+CD25+	Mean	7.04	8.170	7.53	8.210	6.15	8.170	8.23	12.240	5.13	7.190	7.45	7.560	0.9705	0.964	0.0312	0.1471	0.1174	0.2198
	SEM	0.948	1.176	0.569	0.474	0.353	0.600	1.702	1.458	0.217	0.375	0.601	0.149	U	U	MW	MW	U	MW
CD4+Tem	Mean	15.96	16.260	31.31	38.370	35.99	41.410	17.52	19.990	23.71	27.640	38.74	45.390	0.0005	0.4866	0.0259	0.0011	0.0179	0.4004
	SEM	1.107	2.204	2.947	3.366	2.149	2.377	1.583	1.836	2.176	2.040	3.228	4.475	U	U	U	U	U	U
CD4+Tcm	Mean	1.75	2.150	1.91	1.670	2.21	2.540	2.24	2.430	2.27	1.820	0.80	1.230	0.2347	0.0708	0.0693	0.042	0.5749	0.0225
	SEM	0.158	0.426	0.174	0.185	0.289	0.404	0.278	0.264	0.313	0.183	0.275	0.449	U	W	U	MW	U	MW
CD8+	Mean	40.020	38.880	41.010	41.410	37.380	34.900	36.360	23.630	34.090	35.580	37.580	41.410	0.4506	0.0692	0.465	0.8458	0.1318	0.3676
	SEM	1.517	1.191	3.370	3.044	3.333	3.132	0.871	1.558	2.240	1.763	1.968	1.291	W	MW	U	U	U	MW
CD8+Tnv	Mean	13.070	22.620	16.000	19.660	9.886	21.170	24.770	30.631	17.800	26.860	1.595	7.364	0.71	0.8069	0.4278	0.0002	0.3463	0.0005
	SEM	1.982	6.109	3.112	4.584	1.183	3.786	5.086	4.694	5.400	4.483	1.163	0.151	U	U	MW	MW	MW	W
CD8+CD25+	Mean	6.209	2.632	3.180	3.459	2.608	1.533	15.380	7.874	1.571	0.510	1.881	0.698	0.3355	0.1402	0.0052	0.9364	<0.0001	0.011
	SEM	3.233	1.226	0.463	0.900	0.310	0.213	2.718	1.550	0.248	0.062	0.560	0.207	MW	MW	W	MW	MW	MW
CD8+Tem	Mean	18.670	16.080	38.650	44.290	33.090	37.830	19.230	16.380	31.810	30.260	43.370	52.180	0.0002	0.3473	0.0053	0.0011	0.038	0.0383
	SEM	1.426	1.575	4.822	5.143	3.864	3.983	1.589	1.609	4.002	3.082	5.290	4.630	W	U	U	U	U	U
CD8+Tcm	Mean	1.163	1.538	2.357	5.060	1.860	4.573	2.198	2.155	6.716	4.802	0.757	0.949	0.1856	0.8251	0.0239	0.0041	0.9016	0.0286
	SEM	0.175	0.381	0.671	1.764	0.416	1.083	0.354	0.214	1.346	0.789	0.311	0.584	MW	MW	MW	MW	MW	MW
**Functional activation profile of splenic T cells after in vitro stimulation with *Tc* lysate (Percentages of parent populations)**																			
CD4+Tnv IFNγ	Mean	0.386	0.546	0.369	0.475	0.887	3.022	0.606	0.604	0.525	0.641	2.556	1.378	0.1797	<0.0001	0.6859	0.0045	0.0131	0.0002
	SEM	0.043	0.057	0.036	0.062	0.035	0.146	0.056	0.084	0.038	0.047	0.240	0.344	MW	MW	U	MW	MW	U
CD4+Tnv PRF1	Mean	0.457	0.651	0.484	0.602	0.854	2.623	0.538	0.639	0.461	0.608	2.704	1.017	0.6264	<0.0001	0.6934	0.7128	0.9856	0.029
	SEM	0.025	0.045	0.035	0.871	0.045	0.162	0.039	0.051	0.035	0.068	0.445	0.477	W	U	U	MW	U	MW
CD4+Tnv GZB	Mean	0.586	0.643	0.810	1.214	0.947	2.212	0.460	0.608	0.550	0.431	3.233	2.483	0.002	<0.0001	0.0047	0.0002	<0.0001	0.3676
	SEM	0.048	0.059	0.031	0.104	0.059	0.150	0.022	0.045	0.060	0.031	0.955	1.042	U	U	U	MW	W	MW
CD4+Tem IFNγ	Mean	0.541	0.748	0.761	0.840	0.676	0.845	0.639	0.677	0.614	0.807	0.706	0.580	0.5128	0.9709	0.0524	0.0043	0.6981	0.0347
	SEM	0.061	0.120	0.056	0.078	0.023	0.077	0.058	0.059	0.036	0.032	0.048	0.065	U	U	U	U	W	U
CD4+Tem PRF1	Mean	0.481	0.799	0.384	0.481	0.422	0.520	0.528	0.723	0.311	0.526	0.355	0.452	0.0232	0.6577	0.1389	0.4128	0.8088	0.3418
	SEM	0.037	0.108	0.030	0.072	0.024	0.046	0.083	0.116	0.020	0.069	0.036	0.049	U	U	MW	MW	MW	U
CD4+Tem GZB	Mean	0.875	1.003	1.817	2.045	0.550	1.435	0.568	0.719	0.727	0.821	0.833	1.013	0.0552	0.5387	0.2323	0.1943	0.0004	0.012
	SEM	0.035	0.089	0.186	0.336	0.050	0.066	0.029	0.042	0.033	0.057	0.028	0.157	MW	MW	U	U	MW	U
CD4+Tcm IFNγ	Mean	0.539	0.917	0.474	1.343	1.634	4.396	0.517	3.066	0.5165	1.460	3.913	2.552	0.3244	<0.0001	0.0207	0.0879	0.9101	0.5808
	SEM	0.139	0.184	0.067	0.481	0.507	0.285	0.063	0.236	0.0913	0.621	0.563	1.369	MW	MW	MW	MW	MW	W
CD4+Tcm PRF1	Mean	0.764	1.400	0.420	1.562	5.388	4.396	0.813	3.066	0.706	1.460	3.696	2.552	0.8201	0.0004	0.0152	0.2098	0.2752	0.019
	SEM	0.102	0.333	0.040	0.469	0.277	0.307	0.153	0.424	0.063	0.642	0.547	0.752	MW	MW	MW	MW	MW	U

CD4+Tcm	Mean	0.977	1.449	0.989	3.678	0.683	5.291	0.428	2.438	0.7475	1.465	3.893	3.098	0.0047	0.4176	0.0172	0.1394	0.0033	0.2238
GZB	SEM	0.103	0.059	0.071	0.683	0.051	1.247	0.037	0.342	0.0677	0.669	0.581	0.730	MW	MW	MW	MW	MW	U
CD8+Tnv	Mean	0.295	0.567	0.38	0.390	2.27	0.51	0.44	0.480	0.324	0.300	6.81	1.23	0.0145	0.1108	0.0019	0.0367	0.2902	0.0823
IFNγ	SEM	0.034	0.078	0.022	0.055	0.244	0.083	0.045	0.062	0.016	0.021	1.207	0.328	MW	MW	MW	W	MW	W
CD8+Tnv	Mean	0.388	0.613	0.40	0.580	2.44	0.340	0.37	0.490	0.449	0.410	6.12	1.790	0.7934	0.0567	0.2103	0.0393	0.1354	0.0335
PRF1	SEM	0.038	0.052	0.031	0.104	0.208	0.054	0.032	0.058	0.028	0.034	1.163	0.500	W	W	U	W	W	W
CD8+Tnv	Mean	0.535	0.586	0.66	1.160	2.88	0.52	0.48	0.420	0.424	0.430	4.89	1.170	0.0023	0.0004	0.8576	0.0974	<0.0001	0.0244
GZB	SEM	0.069	0.047	0.050	0.124	0.161	0.063	0.047	0.037	0.027	0.023	1.426	0.365	MW	MW	U	W	W	MW
CD8+Tem	Mean	0.528	0.706	0.60	0.670	0.61	0.750	0.48	0.500	0.493	0.770	0.68	1.100	0.6329	0.1642	0.0017	0.0043	0.2642	0.0061
IFNγ	SEM	0.060	0.123	0.047	0.074	0.037	0.062	0.053	0.037	0.017	0.050	0.037	0.095	MW	MW	U	U	U	U
CD8+Tem	Mean	0.335	0.459	0.28	0.370	0.51	0.350	0.26	0.330	0.242	0.310	0.54	0.620	0.3874	0.8047	0.7665	0.0029	0.4663	0.0058
PRF1	SEM	0.034	0.090	0.022	0.059	0.040	0.036	0.038	0.045	0.012	0.039	0.056	0.089	U	U	U	U	U	U
CD8+Tem	Mean	0.731	0.802	0.61	1.500	0.74	1.400	0.48	0.610	0.785	1.700	1.64	2.280	0.1017	0.7528	0.001	0.1353	0.5732	0.008
GZB	SEM	0.083	0.126	0.070	0.272	0.023	0.082	0.035	0.071	0.070	0.196	0.099	0.350	W	W	W	U	U	W
CD8+Tcm	Mean	0.324	0.648	0.46	0.460	3.59	0.530	0.58	0.490	0.463	0.500	4.33	1.250	0.0792	0.4858	0.4262	0.1816	0.221	0.1983
IFNγ	SEM	0.034	0.076	0.042	0.091	0.264	0.092	0.069	0.105	0.059	0.044	0.619	0.487	MW	MW	MW	W	MW	W
CD8+Tcm	Mean	0.353	0.483	0.46	0.730	3.21	0.430	0.58	1.040	0.334	0.640	4.27	2.130	0.4242	0.0719	0.0196	0.107	0.885	0.0755
PRF1	SEM	0.042	0.067	0.034	0.144	0.185	0.097	0.063	0.136	0.037	0.085	0.928	0.762	MW	MW	U	W	MW	MW
CD8+Tcm	Mean	0.601	0.749	1.56	3.090	2.95	1.310	0.50	0.700	1.267	1.020	4.35	2.250	<0.0001	0.0019	0.0094	0.2987	0.0004	0.2621
GZB	SEM	0.056	0.104	0.130	0.335	0.269	0.161	0.027	0.066	0.146	0.106	0.878	0.931	W	MW	MW	MW	MW	M

Female C57BL/6 mice were inoculated with empty plasmid or nano2/4 vaccine, challenged with *Tc*, mated with males, and euthanized at E12-17 gestation days. The age-matched, non-treated (C), infected (Tc) and vaccinated/infected (VaTc) mice were included as controls. Splenocytes were incubated for 48 h with or without antigenic Tc lysate (TcL) and then labeled with fluorophore-conjugated antibodies. $CD3^+$T cells based on the expression levels of CD4, CD8, CD62L, and CD44 antigens were acquired by flow cytometry and data were analyzed by FlowJo software. Percent frequencies of $CD4^-CD8^-$, CD4+CD8+ T lymphocytes, and $CD4^+$ and $CD8^+$ T cell subsets that exhibited Tnv, Tem, and Tcm phenotypes are shown. The frequencies of IFNγ, PRF1 and GZB producing T subsets are also shown. Mean ± SEM values were derived from duplicate observations per sample (n=6-12 mice per group). Significance was calculated by Students' unpaired t test (U) with or without Welch's correction (W) or Mann-Whitney U test (MW) and p values at <0.05 are underlined. These data are presented in **Supplementary Figure 3.**

Supplementary Table 6. Vaccine mediated protection from *T. cruzi* infection and tissue pathology in pregnant mice.

Parameter	Values	Control (C) n=8	Vaccine (Va) n=6	*T. cruzi* (Tc) n=11	VaTc n=10	Pregnant (P) n=6	VaP n=6	TcP n=10	VaTcP n=6	P values / Statistical test								
										C vs P	C vs Va	C vs Tc	Tc vs VaTc	P vs VaP	P vs TcP	TcP vs VaTcP	Tc vs TcP	VaTc vs VaTcP
Cardiac Tc18SrDNA	Mean	0.710	0.990	7563	37.500	2.158	0.800	264.0	129.7	-	-	-	0.0004	-	-	0.4278	0.0029	0.2238
	SEM	.3.253	0.253	5971	31.600	0.334	0.319	244.5	111.2				MW			MW	MW	MW
Sk Mus Tc18SrDNA	Mean	0.850	1.738	433286	22236	1.205	1.077	23149	38049	-	-	-	0.7209	-	-	0.8749	0.6965	0.9497
	SEM	0.520	0.677	418400	11921	0.567	0.182	15748	36352				MW			MW	MW	MW
Cardiac, % Nuclei	Mean	6.660	6.750	10.730	6.950	6.560	6.750	7.670	6.260	0.8140	0.8628	0.0032	0.0088	0.1994	0.1471	0.0559	0.0048	0.3918
	SEM	0.280	0.000	1.060	0.710	0.300	0.000	1.000	0.350	U	U	W	U	U	MW	MW	MW	U
Cardiac, % Tissue tear	Mean	10.700	17.000	24.800	16.100	11.200	15.500	19.000	11.700	0.6356	0.0001	<0.0001	0.0009	0.0038	<0.0001	<0.0001	0.0159	0.0311
	SEM	0.700	0.658	1.830	1.167	0.698	0.911	0.773	1.316	U	U	W	U	U	U	U	W	U
Cardiac, % Fibrosis	Mean	14.700	14.000	25.100	17.600	11.800	11.400	18.000	15.700	0.1468	0.5367	0.0012	0.0146	0.7916	0.0019	0.1687	0.0086	0.4645
	SEM	1.165	0.547	2.111	1.778	1.422	0.310	0.931	1.420	U	U	U	U	W	U	U	W	U
SkM, % Nuclei	Mean	1.990	2.000	15.400	7.920	2.440	2.420	10.000	5.660	0.0418	0.3582	<0.0001	<0.0001	0.9272	<0.0001	0.0002	0.0001	0.0106
	SEM	0.180	0.000	0.860	0.600	0.090	0.190	1.000	0.320	W	U	W	U	U	W	W	U	U
SkM, % Tissue tear	Mean	12.400	18.700	37.300	23.100	15.600	15.800	31.200	24.900	0.0800	0.0038	<0.0001	0.0104	0.8892	<0.0001	0.0405	0.0545	0.3121
	SEM	1.050	1.573	2.956	1.034	1.172	1.010	2.005	1.347	U	U	MW	MW	U	U	U	MW	U
SkM, % Fibrosis	Mean	12.200	9.140	33.000	22.500	8.510	11.900	20.800	16.900	0.1250	0.2004	<0.0001	0.0257	0.0433	0.0002	0.1520	0.0055	0.0269
	SEM	2.021	0.999	3.652	1.553	0.962	1.094	1.769	1.503	W	W	U	W	U	U	U	U	U

Female C57BL/6 mice were immunized with nano2/4, infected with *Tc*, and mated with males as described in Fig.1A. Control females were similarly treated but not mated. To measure parasite burden, total DNA was isolated from heart and skeletal muscle (SkM) tissues of control and pregnant groups and submitted to real-time qPCR amplification of *Tc*18SrDNA sequence (normalized to murine Gapdh). For histological analysis, paraffin-embedded 5-μm tissue sections were stained with H&E. Thresholding methods with ImageJ software were applied to calculate the percent nuclei as a measure of inflammatory infiltrate and tissue tear as a measure of tissue necrosis. Tissue sections were stained with Masson's Trichrome and blue colored collagen deposition as a measure of fibrosis was measured as described in methods. At least two non-consecutive tissue sections per mouse per tissue were scored in 9-10 microscopic fields to calculate the mean-values ± SEM (n=6-11 mice per group). Significance was calculated by Students' unpaired t test (U) with or without Welch's correction (W) or Mann-Whitney U test (MW) and p values at <0.05 are underlined. These data are presented in **Figure 5 and Figure 6.**

Supplementary Table 7. Placental T cell response to *T. cruzi* infection in pregnant mice (± nano2/4).

Parameter	Values	Pregnant (P) n=6	Vaccine (Va) P n=6	*T. cruzi* (Tc) P n=9	VaTcP n=6	Stats: P values / Test applied			
						P vs VaP	P vs TcP	TcP vs VaTcP	VaP vs VaTcP
**Ex vivo phenotypic profile of placental T cells (Percentages of parent populations)**									
CD4-CD8-T	Mean	63.570	63.720	47.650	21.500	0.9783	0.0174	0.0006	<0.0001
	SEM	2.670	4.664	4.386	2.710	U	U	U	U
CD4+CD8+	Mean	4.110	16.970	2.910	43.300	0.3939	0.7756	0.0004	0.0649
	SEM	1.640	6.172	0.700	8.000	MW	MW	MW	MW
CD4+	Mean	19.600	11.700	24.400	32.500	0.0048	0.1560	0.2998	0.0264
	SEM	1.890	1.106	2.290	6.710	U	U	W	W
CD4+Tnv	Mean	47.000	20.580	19.700	7.890	0.0068	0.0012	0.0123	0.0127
	SEM	6.850	3.671	3.010	2.030	U	U	U	U
CD4+Treg	Mean	4.970	9.170	9.810	8.010	0.0517	0.0002	0.2538	0.6097
	SEM	0.290	1.654	0.780	1.440	W	W	U	U
CD4+Tem	Mean	13.830	16.940	22.100	20.230	0.5829	0.1072	0.5414	0.2802
	SEM	4.880	2.500	2.720	1.430	U	U	U	U
CD4+Tcm	Mean	22.330	31.190	17.760	35.710	0.1034	0.4252	0.0084	0.4179
	SEM	4.120	2.738	3.600	4.590	U	U	U	U
CD8+	Mean	12.740	7.640	24.990	2.700	0.0087	0.0070	0.0004	0.0411
	SEM	1.440	1.753	5.440	1.200	MW	MW	MW	MW
CD8+Tnv	Mean	51.760	11.680	9.630	22.800	0.0001	0.0004	0.0044	0.0008
	SEM	1.170	1.899	2.610	1.410	U	MW	MW	U
CD8+Treg	Mean	2.040	3.010	1.330	2.640	0.3774	0.3065	0.0006	0.6857
	SEM	0.620	0.842	0.180	0.240	U	W	U	W
CD8+Tem	Mean	7.810	18.800	37.880	24.820	0.0022	0.0045	0.1494	0.1797
	SEM	0.980	1.809	7.720	3.310	W	MW	W	MW
CD8+Tcm	Mean	11.420	20.450	12.490	27.680	0.1361	0.6989	0.0004	0.2514
	SEM	2.220	5.109	1.651	3.024	U	U	U	U
**Ex vivo functional activation profile of placental T cells (Percentages of parent population)**									
CD4+Treg IL10	Mean	5.020	4.170	6.070	4.950	0.6683	0.5739	0.6010	0.8182
	SEM	1.440	1.290	1.130	0.920	U	U	MW	MW
CD4+Treg IFNγ	Mean	1.650	3.570	0.450	4.870	0.5671	0.1532	0.9999	0.3723
	SEM	0.800	2.270	0.090	2.980	MW	MW	MW	MW
CD4+Treg TNFα	Mean	2.810	17.300	4.120	4.360	0.5887	0.9546	0.6889	0.2403
	SEM	0.440	11.600	1.330	1.730	MW	MW	MW	MW
CD4+Tem IL10	Mean	3.560	1.530	0.210	1.510	0.1126	0.011	0.0937	0.2424
	SEM	1.020	1.528	0.090	0.630	MW	MW	MW	MW
CD4+Tem IFNγ	Mean	0.000	0.000	0.070	0.040	0.9999	0.2286	0.6440	0.9999
	SEM	0.000	0.000	0.040	0.040	MW	MW	MW	MW
CD4+Tem TNFα	Mean	0.790	0.430	0.160	3.480	0.5455	0.2711	0.0060	0.0584
	SEM	0.370	0.310	0.080	2.110	MW	MW	MW	MW
CD4+Tcm IL10	Mean	1.650	2.290	1.110	0.640	0.5541	0.0731	0.7818	0.5671
	SEM	0.390	1.333	0.780	0.360	MW	MW	MW	MW
CD4+Tcm IFNγ	Mean	0.140	2.160	0.000	0.080	0.3182	0.1429	0.4000	0.1818
	SEM	0.090	2.160	0.000	0.080	MW	MW	MW	MW
CD4+Tcm TNFα	Mean	3.895	0.180	1.630	0.420	0.0022	0.0316	0.4294	0.4805
	SEM	1.448	0.093	0.790	0.180	MW	MW	MW	U
CD8+Treg IL10	Mean	8.703	1.411	2.977	0.758	0.0368	0.2326	0.0933	0.2424
	SEM	4.038	0.453	1.231	0.758	MW	MW	MW	MW
CD8+Treg IFNγ	Mean	4.577	0.000	0.442	0.758	0.0606	0.1045	0.8681	0.9999
	SEM	2.318	0.000	0.291	0.758	MW	MW	MW	MW
CD8+Treg TNFα	Mean	20.890	1.063	4.524	0.000	0.0022	0.0008	0.4224	0.0709
	SEM	3.580	0.388	1.525	0.000	MW	MW	MW	MW
CD8+Tem IL10	Mean	2.974	0.063	0.243	0.000	0.0152	0.0286	0.0937	0.2424
	SEM	1.543	0.063	0.175	0.000	MW	MW	MW	MW
CD8+Tem IFNγ	Mean	2.796	0.000	0.009	0.000	0.0606	0.0110	0.9999	0.9999
	SEM	1.492	0.000	0.009	0.000	MW	MW	MW	MW
CD8+Tem TNFα	Mean	1.136	0.198	0.130	0.000	0.0537	0.0937	0.1362	0.0872
	SEM	0.419	0.093	0.057	0.000	U	MW	MW	MW
CD8+Tcm IL10	Mean	1.677	51.480	0.093	8.333	0.0290	0.0002	0.7429	0.0087
	SEM	0.226	16.430	0.093	8.333	W	MW	MW	MW
CD8+Tcm IFNγ	Mean	1.410	0.207	0.091	0.000	0.1818	0.1363	0.9999	0.9999

	SEM	0.872	0.207	0.091	0.000	MW	MW	MW	MW
CD8+Tcm TNFα	Mean	1.127	0.000	0.093	0.000	0.0606	0.0200	0.9999	0.9999
	SEM	0.544	0.000	0.093	0.000	MW	MW	MW	MW

Female C57BL/6 mice were given nano2/4 vaccine, infected with *Tc* at 21 days post-second vaccine dose, mated with males at 7 days post-infection, and euthanized at E12-17 gestation days. Placenta from pregnant mice that were non-infected (P), vaccinated (VaP), *Tc*-infected (TcP), or vaccinated/infected (VaTcP) were obtained. Single cell suspensions of placental cells were labeled with fluorophore conjugated antibodies and analyzed by flow cytometry equipped with FlowJo software. Bar graphs show the percent frequencies of $CD4^-CD8^-$ and $CD4^+CD8^+$ T lymphocytes, and CD4 and CD8 T cell subsets that exhibited Treg, Tnv, Tem, or Tcm phenotypes and produced or IL10, IFNγ, and TNFα cytokines. Mean ± SEM values were derived from duplicate observations per placental sample (n=placentas from 6-9 mice per group). Significance was calculated by Students' unpaired t test (U) with or without Welch's correction (W) or Mann-Whitney U test (MW) and p values <0.05 are underlined. These data are plotted in **Figure 7.**

Supplementary Table 8. Vaccine efficacy in improving the placental/fetal outcomes in infected pregnant mice.

Parameter	Values	Pregnant (P)	Vaccine (Va)P	*T. cruzi* (Tc)P	VaTcP	Stats: P values / T test applied				
						P vs VaP	P vs TcP	VaP vs TcP	VaP vs VaTcP	TcP vs VaTcP
Breeding time (days)	Mean	12.83	11.17	30.10	20.17	0.815	0.999	0.937	0.348	0.999
	SEM	5.53	4.19	16.08	8.12	U	MW	MW	U	MW
% Variance in placenta weight	Mean	13.09	16.76	23.57	11.73	0.212	0.024	0.199	0.0876	0.013
	SEM	1.19	2.49	3.72	0.94	U	W	U	U	W
% Variance in placenta efficiency	Mean	19.23	18.81	36.99	10.57	0.930	0.271	0.088	0.1238	0.0008
	SEM	1.34	4.40	9.370	1.280	W	MW	W	W	MW
# Fetuses per dam	Mean	8.00	8.50	6.20	7.33	0.729	0.092	0.026	0.0931	0.799
	SEM	0.680	0.428	0.860	0.560	MW	MW	MW	MW	MW
% Resorbed fetuses	Mean	9.17	3.70	17.14	2.38	0.549	0.092	0.026	0.0931	0.799
	SEM	3.21	3.70	9.71	2.38	U	MW	MW	MW	MW
Placenta Tc18SrDNA	Mean	1.12	1.53	499	1751	0.317	0.0001	0.0001	0.0001	0.0081
	SEM	0.180	0.270	284	833	MW	MW	MW	MW	MW
Fetus Tc18SrDNA	Mean	0.95	1.02	12.11	3.00	0.628	0.277	0.642	0.0803	0.268
	SEM	0.250	0.214	10.49	0.65	MW	MW	MW	MW	MW
Placenta, % Nuclei	Mean	14.600	12.800	20.90	13.30	0.152	<0.0001	<0.0001	0.166	<0.0001
	SEM	0.780	0.220	0.230	0.320	MW	MW	MW	U	MW
Placenta, % Tissue tear	Mean	32.400	30.500	32.90	29.80	0.019	0.622	0.005	0.302	0.001
	SEM	0.670	0.476	0.703	0.556	U	U	U	U	U
Placenta, % Fibrosis	Mean	54.80	54.00	57.00	52.60	0.399	0.048	0.0095	0.214	0.0013
	SEM	0.610	0.674	0.910	0.909	U	W	U	U	U

C57BL/6 female mice were immunized with nano2/4, infected with *Tc* at 21 days post 2nd vaccine dose, mated with males at 7 days post-infection, and euthanized at 12-17 gestation days. Breeding time post-pairing, percent variation in placental weight and placental efficiency, frequency of full-term and resorbed fetuses was recorded. Placental and fetal parasite burden was evaluated by real-time qPCR measurement of *Tc*18SrDNA sequence (normalized to murine Gapdh). For histological analysis, paraffin-embedded 5-µm tissue sections were stained with H&E. Thresholding methods with ImageJ software were applied to calculate the percent nuclei as a measure of inflammatory infiltrate and tissue tear as a measure of necrosis. Tissue sections were stained with Masson's Trichrome and blue colored collagen deposition as a measure of fibrosis was measured as described in methods. At least two non-consecutive placental tissue sections per sample were scored in 9-10 microscopic fields. All data are presented as mean values ± SEM. Breeding time post-pairing, percent variation in placental weight, placental efficacy, number of fetuses per dam and resorbed number of fetuses were calculated from n=6 (P), n=6 (VaP), n=10 (TcP), and n=6 (VaTcP) females. For parasite burden, # females / # placentas (or fetuses) used were n=6/32 (P), 6/37 (VaP), 10/39 (TcP), 6/31 (VaTcP). For histology, # females / # placentas used were n=6/28 (P), n=6/37 (VaP), n=10/36 (TcP), and n=6/30 (VaTcP). Data are presented as mean-values ± SEM. Significance was calculated by Students' unpaired t test (U) with or without Welch's correction (W) or Mann-Whitney U test (MW) and p values at <0.05 are underlined. Data are shown in **Figure 8.**

a

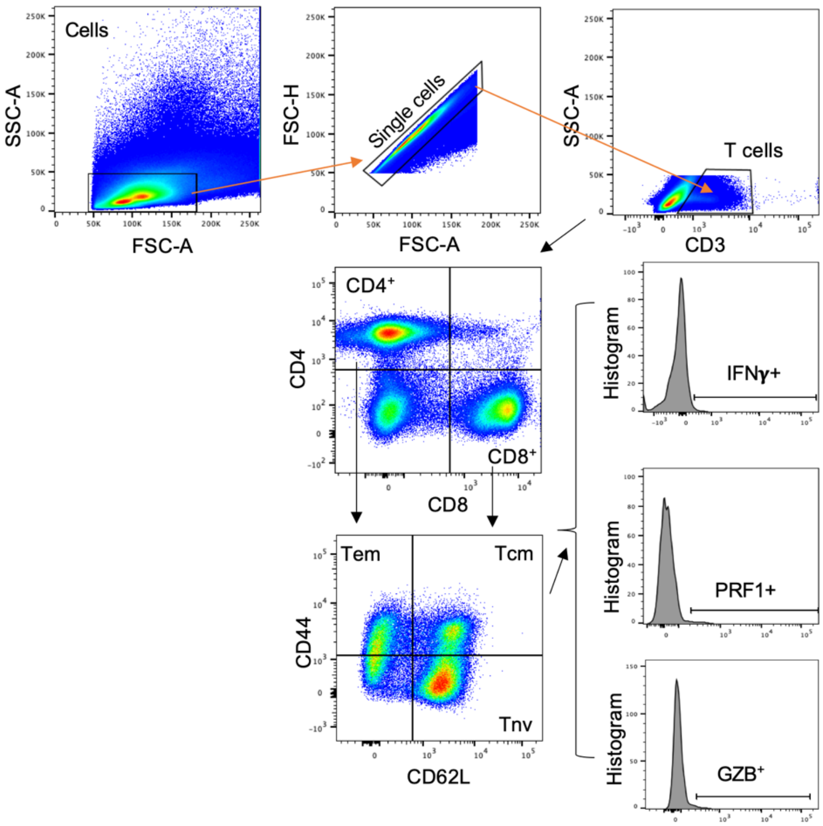
Cells
SSC-A
FSC-A
Single cells
FSC-H
FSC-A
T cells
SSC-A
CD3
CD4⁺
CD4
CD8
CD8⁺
Tem
Tcm
Tnv
CD44
CD62L
Histogram
IFNγ+
Histogram
PRF1+
Histogram
GZB⁺

b

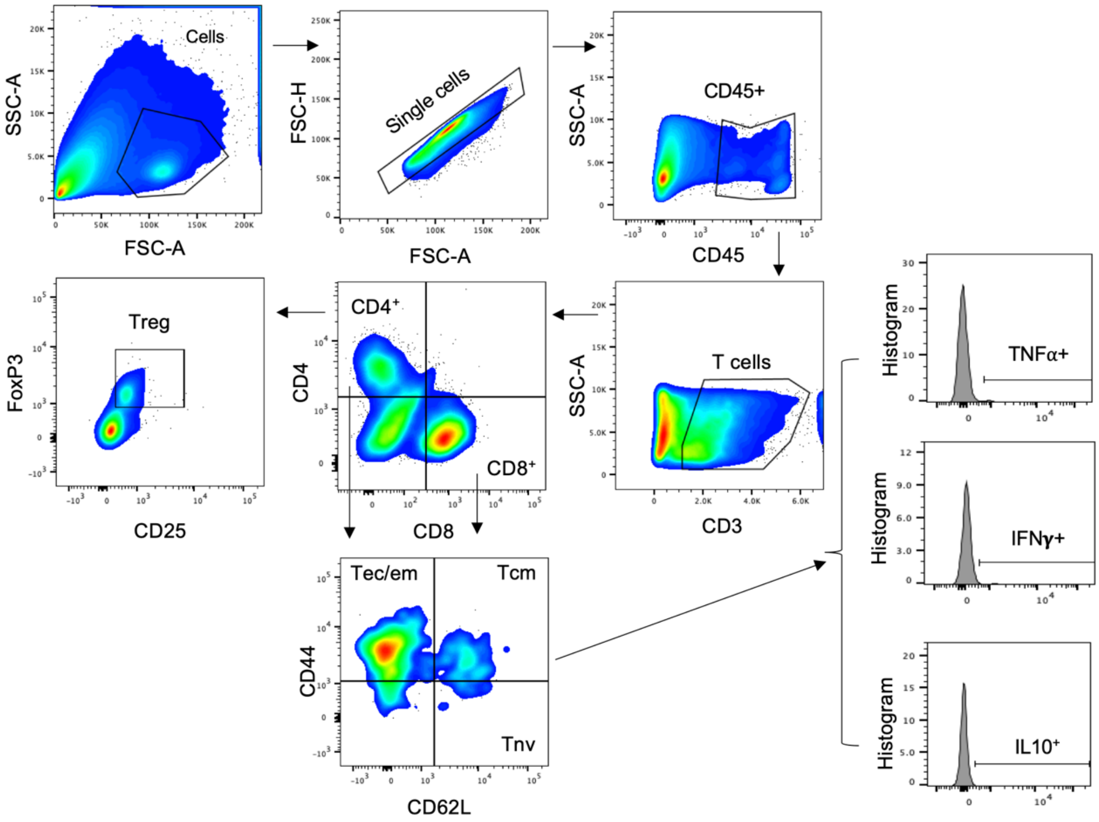
Cells
SSC-A
FSC-A
Single cells
FSC-H
FSC-A
CD45+
SSC-A
CD45
Treg
FoxP3
CD25
CD4⁺
CD4
CD8
CD8⁺
T cells
SSC-A
CD3
Tec/em
Tcm
Tnv
CD44
CD62L
Histogram
TNFα+
Histogram
IFNγ+
Histogram
IL10⁺

Supplementary Figure 1. Schematics of gating strategy for the analysis of splenic T cell subpopulations by flow cytometry. Female mice were immunized with nano2/4, infected with *Tc*, mated, and euthanized at day 19-24 post infection (=E12-17 gestation days), as described in Fig.1A. **(a)** Splenic cells from all mice (*n* ≥ 5 per group) were stained with 10-color fluorochromes. Lymphocytes were captured from acquired cells using a LSRII Fortessa. After gating for live lymphocytes in forward and side scatter, cell aggregates/doublets were ignored and $CD3^{+}$ FMO control was used to gate CD3+, single T lymphocytes from all groups of mice. $CD3^{+}$T cells based on the expression levels of CD4, CD8, CD62L, and CD44 antigens were acquired and analyzed by FlowJo software. The CD4+ and CD8+ effector/effector memory (Tem: CD62L-CD44+), central memory (Tcm: CD62L+CD44+) and naïve (Tnv: CD62L+CD44-) subsets were analyzed for the expression levels of IFNγ, Perforin (PRF1) and Granzyme B (GZB). **(b)** Placental cells from all pregnant mice (*n* ≥ 5 per group) were gated for CD45+ CD3+ single cell lymphocytes and phenotypic subsets were generated by FlowJo analysis as above. The T cell subsets were analyzed for the median fluorescence intensity of IFNγ, TNFα, and IL10. The CD3+CD4+T cells were also examined for the frequency of FoxP3+CD25+ regulatory (Treg) subpopulation.

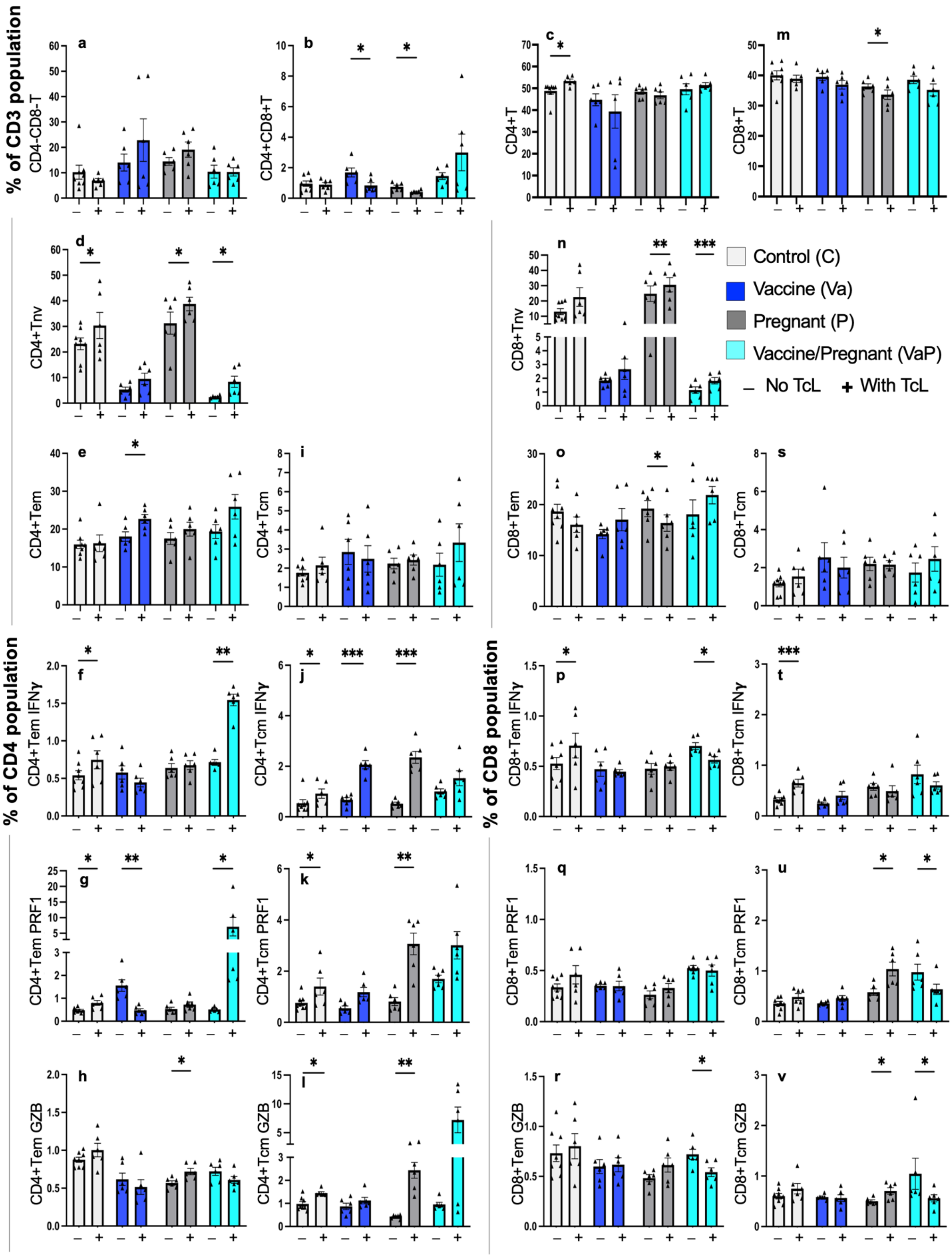

% of CD3 population
% of CD4 population
% of CD8 population
CD4-CD8-T
CD4+CD8+T
CD4+T
CD8+T
CD4+Tnv
CD8+Tnv
CD4+Tem
CD4+Tcm
CD8+Tem
CD8+Tcm
CD4+Tem IFNγ
CD4+Tcm IFNγ
CD8+Tem IFNγ
CD8+Tcm IFNγ
CD4+Tem PRF1
CD4+Tcm PRF1
CD8+Tem PRF1
CD8+Tcm PRF1
CD4+Tem GZB
CD4+Tcm GZB
CD8+Tem GZB
CD8+Tcm GZB
Control (C)
Vaccine (Va)
Pregnant (P)
Vaccine/Pregnant (VaP)
– No TcL
+ With TcL

Supplementary Figure 2. Recall response of vaccine induced T cells to second antigenic stimulation in pregnant mice. C57BL/6 female mice were given empty plasmid or nano2/4 vaccine, mated with males at 28 days post-vaccination, and euthanized at E12-17 gestation days. Age-matched, non-vaccinated and vaccinated (but not mated) mice were included. Splenocytes were *in vitro* stimulated for 48 h with or without antigenic *Tc* lysate (TcL). Cells were then labeled with fluorophore-conjugated antibodies and analyzed by flow cytometry. Shown are percent frequencies of $CD4^{-}CD8^{-}$ **(a)**, CD4+CD8+ **(b)** T lymphocytes, and $CD4^{+}$ **(c-l)** and $CD8^{+}$ (**m-v)** T cell subsets that exhibited Tnv (d, n), Tem (e, o), and Tcm (i, s) phenotypes. Frequencies of IFNγ (f, j, p, t), PRF1 (g, k, q, u) and GZB (h, l, r, v) expressing Tem and Tcm subsets are also shown. Each mouse value is presented by a triangle and mean ± SEM values derived from duplicate observations per sample are plotted (n=6-8 per group). Significance was calculated by Students' unpaired t test (or paired t test for recall response) with or without Welch's correction or Mann-Whitney U test (or Wilcoxon test for recall response) and *p* values of <0.05, <0.01, and <0.001 are annotated with one, two, and three symbols, respectively. Horizontal bar indicates the compared groups. Detailed data are presented in **Supplementary Table 3.**

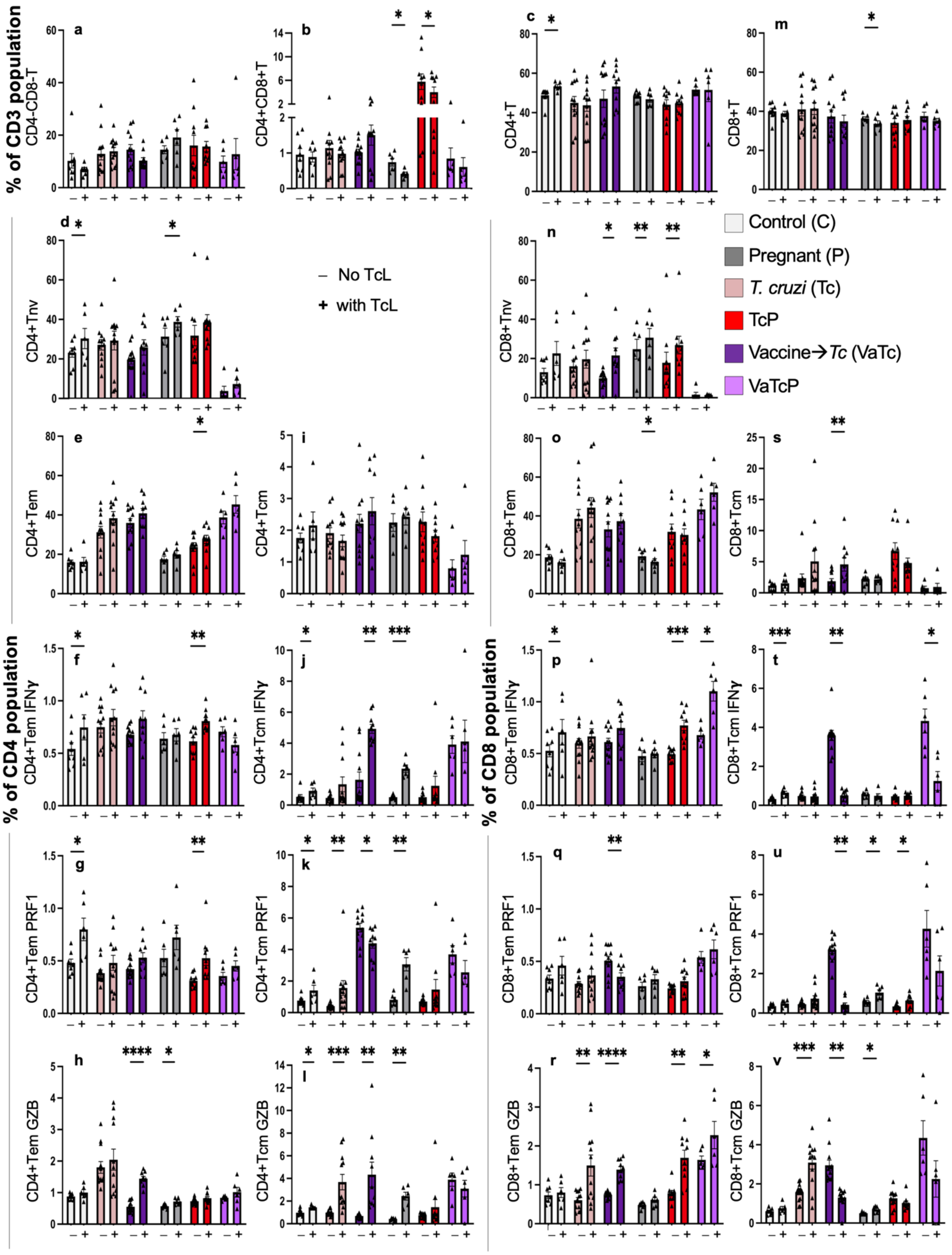

% of CD3 population
% of CD4 population
% of CD8 population
CD4-CD8-T
CD4+CD8+T
CD4+T
CD8+T
CD4+Tnv
CD8+Tnv
CD4+Tem
CD4+Tcm
CD8+Tem
CD8+Tcm
CD4+Tem IFNγ
CD4+Tcm IFNγ
CD8+Tem IFNγ
CD8+Tcm IFNγ
CD4+Tem PRF1
CD4+Tcm PRF1
CD8+Tem PRF1
CD8+Tcm PRF1
CD4+Tem GZB
CD4+Tcm GZB
CD8+Tem GZB
CD8+Tcm GZB
– No TcL
+ with TcL
Control (C)
Pregnant (P)
T. cruzi (Tc)
TcP
Vaccine→Tc (VaTc)
VaTcP

Supplementary Figure 3. Recall response of splenic T cells of vaccinated and infected pregnant mice to in vitro stimulation with parasite antigens*.* C57BL/6 female mice were given empty plasmid or nano2/4 vaccine, challenged with *Tc*, mated with males, and euthanized at E12-17 gestation days. Age-matched, non-treated (C), infected (Tc) and vaccinated/infected (VaTc) mice that were not mated were included as controls. Splenocytes were incubated for 48 h with or without TcL for antigenic stimulation, labeled with fluorophore-conjugated antibodies and analyzed by flow cytometry. Percent frequencies of $CD4^{-}CD8^{-}$ **(a)**, CD4+CD8+ **(b)** T lymphocytes, and $CD4^{+}$ **(c-l)** and $CD8^{+}$ (**m-v)** T cell subsets that exhibited Tnv (d, n), Tem (e, o), and Tcm (i, s) phenotypes are shown. The frequencies of IFNγ (f, j, p, t), PRF1 (g, k, q, u) and GZB (h, l, r, v) producing Tem and Tcm subsets are also shown. Each mouse value is presented by a triangle and mean ± SEM values derived from duplicate observations per sample are plotted. Sample size for TcL untreated mice: C (n=8), Tc (n=12), VaTc (n=12), P (n=6), TcP (n=10), VaTcP (n=6). Sample size for TcL-treated mice: C (n=6), *Tc* (n=12), VaTc (n=10), P (n=6), TcP (n=10), VaTcP (n=6). Significance was calculated by Students' unpaired t test (or paired t test for recall response) with or without Welch's correction or Mann-Whitney U test (or Wilcoxon test for recall response) and *p* values of <0.05, <0.01, <0.001, and <0.0001 are annotated with one, two, three, and four symbols, respectively. Horizontal bar indicates the compared groups. Detailed data are presented in **Supplementary Table 5.**
